# Activation of the HSP27-AKT axis contributes to gefitinib resistance in non-small cell lung cancer cells independent of EGFR mutations

**DOI:** 10.1007/s13402-022-00696-3

**Published:** 2022-08-05

**Authors:** Seul-Ki Choi, Minsuh Kim, Haeseung Lee, Youngjoo Kwon, Hyuk-Jin Cha, Se Jin Jang, Younghwa Na, Yun-Sil Lee

**Affiliations:** 1 Graduate School of Pharmaceutical Sciences, Ewha Womans University, 52, Ewhayeodae-gil, Seodaemun-gu, Seoul, 120-720 Republic of Korea; 2 Asan Center for Cancer Genome Discovery, Asan Institute for Life Sciences, Seoul, 05505 Republic of Korea; 3 College of Pharmacy, Pusan National University, Busan, 46241 Republic of Korea; 4 College of Pharmacy, Seoul National University, Seoul, 08826 Republic of Korea; 5 College of Pharmacy, CHA University, 120, Haeryong-ro, Pocheon, 487-010 Republic of Korea

**Keywords:** Non-small cell lung cancer (NSCLC), Heat shock protein 27 (HSP27), EGFR-TKI resistance, AKT activation

## Abstract

**Purpose:**

Although epidermal growth factor receptor (EGFR)-activating mutations in non-small cell lung cancer (NSCLC) usually show sensitivity to first-generation EGFR-tyrosine kinase inhibitors (TKIs), most patients relapse because of drug resistance. Heat shock protein 27 (HSP27) has been reported to be involved in the resistance of EGFR-TKIs, although the underlying mechanism is unclear. Here, we explore the mechanisms of HSP27-mediated EGFR TKI resistance and propose novel therapeutic strategies.

**Methods:**

To determine the mechanism of HSP27 associated gefitinib resistance, differences were assessed using gefitinib-sensitive and -resistant NSCLC cell lines. *In vivo* xenograft experiments were conducted to elucidate the combinatorial effects of J2, a small molecule HSP27 inhibitor, and gefitinib. Analyses of human NSCLC tissues and PDX tissues were also used for comparison of HSP27 and phosphorylated AKT expression.

**Results:**

Large-scale cohort analysis of NSCLC cases revealed that HSP27 expression correlated well with the incidence of EGFR mutations and affected patient survival. Increased pAKT and HSP27 was observed in gefitinib-resistant cells compared with gefitinib-sensitive cells. Moreover, increased phosphorylation of HSP27 by gefitinib augmented its protein stability and potentiated its binding activity with pAKT, which resulted in increased gefitinib resistance. However, in gefitinib-sensitive cells, stronger binding activity between EGFR and HSP27 was observed. Moreover, these phenomena occurred regardless of EGFR mutation including secondary mutations, such as T790M. AKT knockdown switched HSP27-pAKT binding to HSP27-EGFR, which promoted gefitinib sensitivity in gefitinib-resistant cells. Functional inhibition of HSP27 yielded sensitization to gefitinib in gefitinib-resistant cells by inhibiting the interaction between HSP27 and pAKT*.*

**Conclusions:**

Our results indicate that combination of EGFR-TKIs with HSP27 inhibitors may represent a good strategy to overcome resistance to EGFR-TKIs, especially in cancers exhibiting AKT pathway activation.

**Supplementary Information:**

The online version contains supplementary material available at 10.1007/s13402-022-00696-3.

## Introduction

The first-generation epidermal growth factor receptor (EGFR) tyrosine kinase inhibitors (TKIs), gefitinib (Gef) and erlotinib, which target activating mutations such as deletion at exon 19 (delE746_A750) or a point mutation at exon 21 (L858R) were designed to compete reversibly for the ATP binding sites and thus block EGFR-induced downstream signaling activation. These proteins are being extensively investigated in NSCLC treatment [1–5]. However, problems have arisen because almost all patients with dramatic initial responses to the first-generation EGFR-TKIs ultimately showed tumor progression and inevitably became resistant within 6–12 months. EGFR-TKIs may elicit multiple mechanisms of resistance including T790M gatekeeper EGFR mutation, MET amplification, conversion to small cell lung cancer, and PI3KCA mutation [6]. However, additional studies aimed at the mechanisms that contribute to EGFR-TKI resistance and the identification of potential approaches to reverse it are necessary.

First-line sensitivity to EGFR-TKIs in NSCLC has been associated with pre-existent AKT activation, and EGFR-TKIs fail to block AKT signaling in tumor cells that are intrinsically or extrinsically resistant to these drugs [7–9]. Activation of the AKT pathway is a common feature in human cancers and leads to increased cell survival, growth and proliferation [10]. AKT activation is induced through both EGFR-dependent and EGFR-independent pathways, and recent work suggests that the AKT pathway is involved in the acquisition of EGFR-TKI-induced resistance [11].

High levels of heat shock protein 27 (HSP27) have been found in many cancer types, and clinical trials have revealed a relationship between HSP27 and aggressive cancers, metastasis, drug resistance and poor patient outcomes [12]. HSP27 has been found to direct chaperoning interactions with among others DAXX, IkB, caspase 3 and PKC [13–15]. Moreover, HSP27 has been reported to be highly expressed in human NSCLC, suggesting that inhibition of HSP27 in NSCLC may be a good therapeutic strategy. Indeed, functional inhibition of HSP27 using small molecules can sensitize NSCLC cells to radiation or conventional anticancer drugs [16].

HSP27 and AKT can form a complex with p38 mitogen-activated protein kinase (MAPK) [17–19]. HSP27 then binds to AKT and acts as a scaffold protein to permit phosphorylation of AKT (Ser 473) by p38 MAPK to protect against cell death by blocking apoptosis [20, 21], which also affects AKT protein stability [21]. This suggests a link from p38 MAPK to AKT activation via HSP27 in response to apoptotic stress in cancer cells. Previously, we found that zerumbone (ZER) and SW15 can induce altered cross-linking of HSP27 and modify its normal dimerization, which results in a tumor sensitizing effect after treatment with anticancer drugs or radiation [22, 23]. Moreover, J2, as a more potent HSP27 cross-linker than SW15, was found to promote sensitization when combined with conventional anticancer drugs in cell lines that overexpress HSP27 [24].

In this study, we further investigated the involvement of HSP27 in Gef resistance of both EGFR-wild type (wt) and resistant mutant (mut) NSCLC cells via AKT activation. HSP27 inhibition using small molecules like J2 effectively abrogated Gef resistance in NSCLC cells independent of EGFR mutations.

## Materials and methods

### Survival analysis

NSCLC patient data were collected from five independent microarray datasets generated using the Affymetrix Human Genome U133 Plus 2.0 Array. A total of 1,086 raw CEL files was obtained from the Gene Expression Omnibus (GEO) database (http://www.ncbi.nlm.nih.gov/geo) with accession IDs GSE19188, GSE30219, GSE31210, GSE37745 and GSE50081. The CEL files were preprocessed and normalized using the Robust Multichip Average [25] method implemented in the R package ‘affy.’ After filtering out non-cancer or non-NSCLC samples, gene expression profiles for 935 patients with survival information were retained for further analysis. Patients were divided into three groups (high, intermediate, low) for the expression level of the gene of interest (e.g., *HSPB1*, *AKT1* or *EGFR*). For survival analysis, high and low groups were used to test differences in overall survival or relapse-free survival rates via the R package ‘survival.’ Hazard ratio (HR) and *p*-value (P) were computed using Cox proportional hazards regression analysis and log-rank test, respectively.

### Cell culture and transfection

Human NSCLC cell lines NCI-H460, A549, NCI-H1650, NCI-H820, HCC827, PC9 and H1975 were cultured in RPMI-1640 medium supplemented with 10% fetal bovine serum and 1% penicillin–streptomycin in a 37 °C incubator with 5% CO_2_. All media were changed every 2–3 days, and the split ratios were from 1:4 to 1:10 according to the ATCC® descriptions. To establish acquired Gef-resistant PC9 (PC9GR) cells, 1 to 1000 nM Gef was administered to PC9 (Gef-sensitive cells) for 4 months to induce resistance. The culture conditions were the same as that of PC9. Transfections were performed using Lipofectamine 2000 (Invitrogen). Cells were then transfected with the designated plasmids in each experiment using transfection reagent according to the manufacturer's protocol. The plasmids or siRNAs used in this study are as follows: siHSP27 (sc-29350), siAKT (sc-43609), p3xflag-myc-HSP27S15A/S78A/S82A, p3xflag-myc-HSP27S15D/S78D/S82D and p3xflag-myc-AKT1WT.

### Compounds and chemicals

J2 was synthesized as described previously [23, 24]. Gefitinib (ZD1895) (Selekchem, S1025), AZD5363 (Seleckchem, S8019), SC-79 (Seleckchem, S7863) and Uprosertib (GSK2141795) (Seleckchem, S7492) were dissolved in DMSO and diluted in cell culture medium.

### MTT cell viability assay

A 3-(4,5-dimethyl-2-thiazolyl)-2,5-diphenyl-2H-tetrazolium bromide methylthiazolyldiphenyl-tetrazolium bromide (MTT, Sigma-Aldrich, M5655) assay was used as an indirect measure of cell viability (see Supplementary Materials).

### Flow cytometry

Cells were washed twice with PBS, dissociated using trypsin–EDTA, and centrifuged at 13,000 rpm for 3 min at 4 °C. After adding 1 ml PBS and 10 μg/ml propidium iodide to samples in polystyrene round-bottom tubes, flow cytometric analysis was performed using a BD FACS Calibur flow cytometer (BD Bioscience, San Jose, CA, USA).

### Immunoblotting, immunoprecipitation, immunohistochemistry and immunofluorescence assays

Immunoblotting, immunoprecipitation, immunohistochemistry, and immunofluorescence assay were performed as described previously [26, 27] using specific antibodies (see Supplementary Materials).

### Colony formation assay

Cells were seeded in 6-well culture plates at a density of 500 cells/well. Compounds were applied to the cells after seeding. After 10 days or 14 days of incubation with or without various compounds, cells were fixed with 100% methanol for 1 h and stained with 2 ml trypan blue solution (1% [w/v] in PBS) per well. Next, cells were rinsed with tap water and analyzed. Images were taken using a ChemiDoc bio-image analyzer (Bio-Rad, Hercules, CA, USA) and quantified using ImageJ software (NIH, Bethesda, MD, USA). All steps after fixation were performed at room temperature.

### Subcellular fractionation

The cytoplasmic and nuclear fractions of cells were collected using NE-PER Nuclear and Cytoplasmic Extraction reagents (Thermo Scientific, Waltham, MA, USA). Sample preparation was performed using the manufacturer’s protocol (see Supplementary Materials).

### Mouse tumor xenografts

Before initiation, all proposed mouse studies were approved by the Institutional Animal Care and Use Committee (IACUC) of Ewha Womans University (IACUC Approval No: 18–006). Mice were cared for and euthanized according to the standards set forth by the IACUC. Mice were raised in a specific pathogen-free environment under a temperature- and light-controlled regimen with free access to food and water.

A single A549 suspension (1 × 10^7^ cells) was injected subcutaneously into the hind legs of 5-week-old BALB/c nude mice (Orientbio, Seongnam, Gyeonggi-do, Korea), and a NCI-H1650 suspension (1 × 10^7^ cells) was injected into 4-week-old female NOD-SCID mice (Koatech, Pyeongtaek, Gyeonggi-do, Korea). When the tumors reached a minimal volume of 150–200 mm^3^, the mice were treated once every 2 days with J2 (20 mg/kg) and once every 3 days with Uprosertib (2 mg/kg) or Gef (5 mg/kg) by intraperitoneal (i.p.) injection. Tumor volumes were determined according to the formula (L × W^2^)/2, using measurements of tumor length (L) and width (W) with a caliper. Tumors were measured twice weekly and allowed to grow to the indicated volume. At the end of the observation period or when tumors reached 1500 mm^2^, mice were euthanized, and tumor xenografts were paraffin-embedded for immunohistochemistry.

### PDX model from human lung cancer organoids

A patient-derived xenograft (PDX) model from lung cancer organoids (LCOs) AMC-15LT-005 and AMC-15LT-006 was generated as described previously [28] in the Asan Institute for Life Sciences, Asan Medical Center, Korea.

### In situ proximity ligation assay (PLA)

PLA was performed to detect the interactions of HSP27 with pAKT and of HSP27 with EGFR. To visualize the bound antibody pairs, a Duolink Detection Kit (Duo92008) with PLA PLUS and MINUS probes for mouse and rabbit (Sigma-Aldrich) was used according to the manufacturer’s instructions.

### Statistical analysis

Values are displayed as mean plus or minus standard deviation or standard error. Statistical significance was determined using Student’s t-test. *p*-values < *0.05* were considered significant.

## Results

### HSP27 overexpression predicts a poor clinical outcome in NSCLC

**Fig. 1:**
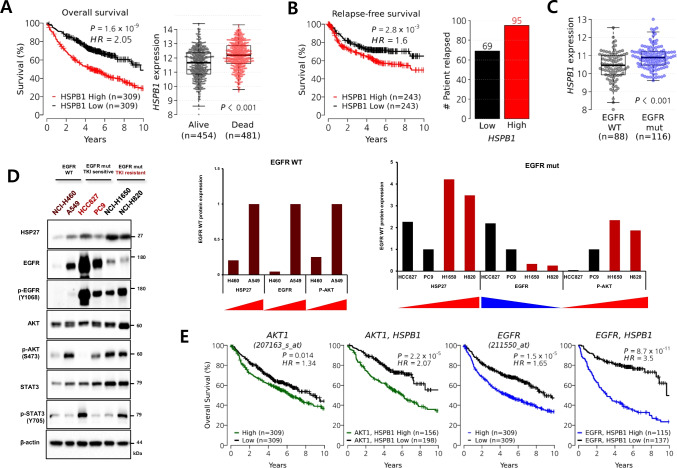

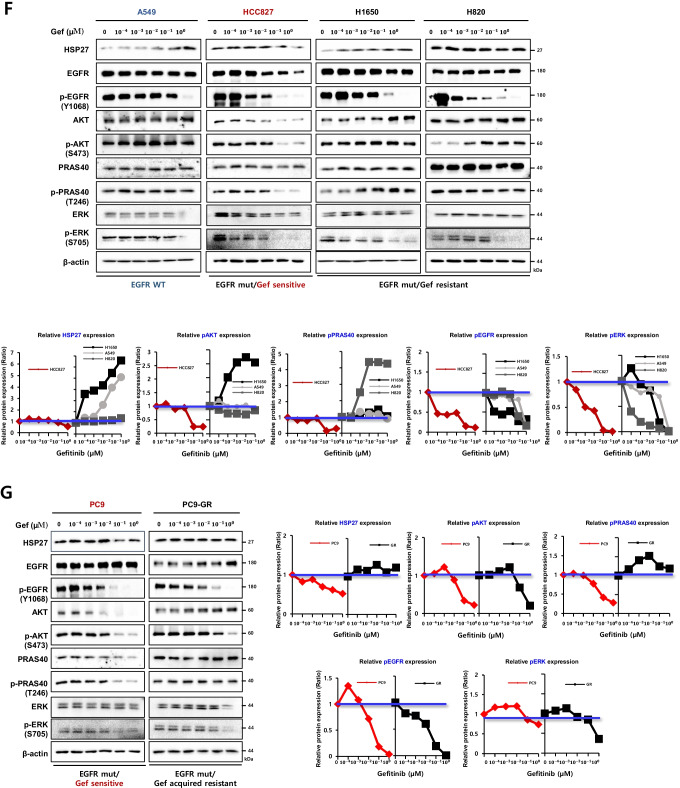
HSP27 overexpression predicts poor clinical outcome in NSCLC. (**A**) Kaplan–Meier plot showing overall survival stratified by *HSPB1* expression level (left) and the difference in *HSPB1* levels of the censored and deceased groups (right) in 935 NSCLC patients. (**B**) Relapse-free survival rate stratified by *HSPB1* expression level (left) and the number of patients with cancer recurrence in *HSPB1*-low and *HSPB1*-high groups. A total of 736 patients with information on relapse-free survival time was used. (**C**) *HSPB1* expression levels grouped by EGFR mutation status of NSCLC patients. Information on *EGFR* alterations was only available in the GSE31210 data set. (**D**) Comparison of protein expression in NSCLC cell lines according to EGFR mutation. Relative band densities are expressed as fold change relative to H460 in EGFR WT cells and PC9 in EGFR mut cells. (**E**) Kaplan–Meier plots showing overall survival rates stratified by the expression levels of *AKT1*, the combination of *AKT1* and *HSPB1*, *EGFR*, and the combination of *AKT1* and *HSPB1* in NSCLC patients. (**F**) Changes in protein expression in NSCLC cells A549, HCC827, H1650, H820 and (**G**) PC9 and PC9-GR cells at the indicated concentration of gefitinib (Gef) treatment (6 h) (upper). Relative band densities are expressed as fold change relative to Gef 0 µM

To assess the prognostic value of HSP27, we investigated transcriptome data from a cohort of 935 NSCLC patients and found that higher expression of *HSPB1* (mRNA of HSP27) was strongly associated with a poor survival, and that the level of *HSPB1* expression was elevated in deceased patients (Supplementary Fig. S1A and Fig. 1A). Consistent with previous reports on the relationship between HSP27 and metastasis [PMID: 23492367], cancer recurrences occurred more frequently in patients overexpressing *HSPB1* (Fig. 1B). Furthermore, EGFR mut NSCLC cases showed higher *HSPB1* expression levels (Fig. 1C).

To clarify the relationship between HSP27 and EGFR activation in a cell system, several NSCLC cell lines were examined. We found that phospho-EGFR (pEGFR) expression was dramatically higher in EGFR mut (HCC827, PC9, NCI-H1650, and NCI-H820) cells than in EGFR wt (NCI-H460 and A549) cells. Even though EGFR mut cells carried similar exon 19 deletions, their sensitivity to Gef was different, i.e., HCC827 and PC9 showed sensitivity to Gef, whereas NCI-H1650 and NCI-H820 cells were resistant to Gef. NCI-H820 cells carry an additional T790M mutation, as well as a delE746_A750. EGFR wt cells showed higher IC_50_ values to Gef (μM level) than Gef-sensitive EGFR mut cells (nM level). However, in Gef-resistant EGFR mut cells such as NCI-H1650 and NCI-H820, IC_50_ values to Gef were similar to those of EGFR wt cells (μM level, Table 1). Gef-resistant EGFR mut cells showed a higher phospho-AKT (pAKT) level at Ser473, which has been reported to be involved in full AKT activation, than Gef-sensitive EGFR mut cells. In the case of EGFR wt cells, A549 cells showed a greater resistance to Gef than NCI-H460 cells, and pAKT expression in A549 cells was found to be well-correlated with Gef resistance. However, phospho-STAT (pSTAT) expression was not correlated with Gef sensitivity in any of the cell lines. Moreover, the expression of HSP27 was higher in EGFR mut Gef-resistant cells than in EGFR mut Gef-sensitive cells. In EGFR wt cells, A549 cells exhibited a higher expression of HSP27 than NCI-H460 cells, which was also well correlated with pAKT expression (Fig. 1D). The expression levels of *AKT1* (mRNA of AKT) and *EGFR* (mRNA of EGFR) were also closely related to a poor prognosis in NSCLC patients. However, high levels of both *AKT1* and *HSPB1* or both *EGFR* and *HSPB1* provided more prognostic information on survival (Fig. 1E), suggesting that HSP27 in combination with AKT or EGFR serves as a strong risk factor for survival in NSCLC patients.

**Table 1:** IC_50_ after gefitinib treatment in NSCLC cell lines

Cell lines	Cancer types	EGFR	EGFR expression	HSP27 expression	Gef (µM) IC_50_	J2 (µM) IC_50_
NCI-H460	Adenocarcinoma	Wild-type	+	+	45.12 ± 2.33 (resistant)	99.27 ± 1.13
A549	Adenocarcinoma	Wild-type	++	++	83.84 ± 9.95 (resistant)	> 100
HCC827	Adenocarcinoma	Mutant-type (Exon19 del)	+++++	+++	0.0022 ± 0.048 (sensitive)	> 100
PC9	Adenocarcinoma	Mutant-type (Exon19 del)	++++	+++	0.061 ± 0.024 (sensitive)	> 100
NCI-H1650	Adenocarcinoma	Mutant-type (Exon19 del)	++	+++++	68.41 ± 2.06 (resistant)	42.44 ± 1.2
NCI-H820	Adenocarcinoma	Mutant-type (Exon19 del, T790M)	+++	+++++	26.3 ± 0.53 (resistant)	48.80 ± 4.38
NCI-H1975	Adenocarcinoma	Mutant-type (L858R, T790M)	+++	++++	54.04 ± 2.19 (resistant)	39.92 ± 0.98
PC9GR	Adenocarcinoma	Mutant-type (Exon19 del)	++++	++++	30.85 ± 2.85 (resistant)	92.03 ± 7.15

Indicated NSCLC cell lines were treated with different concentrations of gefitinib (Gef) or J2 for 24 h and cell viability was analyzed by MTT assay. The table shows the IC_50_ values (mean ± SD) and relative protein expression

Low doses of Gef treatment dose-dependently inhibited pEGFR expression in all cell lines tested, regardless of EGFR wt or mut type. However, Gef treatment of NCI-H1650, NCI-H820 and A549 cells did not inhibit pAKT expression or its downstream pathway such as activation of RPAS40. In Gef-sensitive cells, expression of pAKT, as well as its downstream pathway, were gradually inhibited according to the increase in Gef concentration. Phosphorylated ERK (pERK), another downstream target of EGFR, was activated by Gef in all the cell lines regardless of EGFR wt or mut type (Fig. 1F). However, with respect to pSTAT, the response to Gef did not show any consistency in EGFR wt and mut cells or Gef-sensitive and -resistant cells (Supplementary Fig. S1B and S1C). From the data, we conclude that even though pEGFR expression correlated well with Gef treatment in all the cell lines, high expression of pAKT and HSP27 at basal level or no inhibition of the AKT downstream pathways by Gef treatment was responsible for the Gef resistance. When PC9GR (acquired Gef-resistant PC9 cell line) was compared with PC9 parent cells, similar results were obtained (Fig. 1G).

### HSP27 potentiates AKT activation by direct interactions

**Fig. 2:**
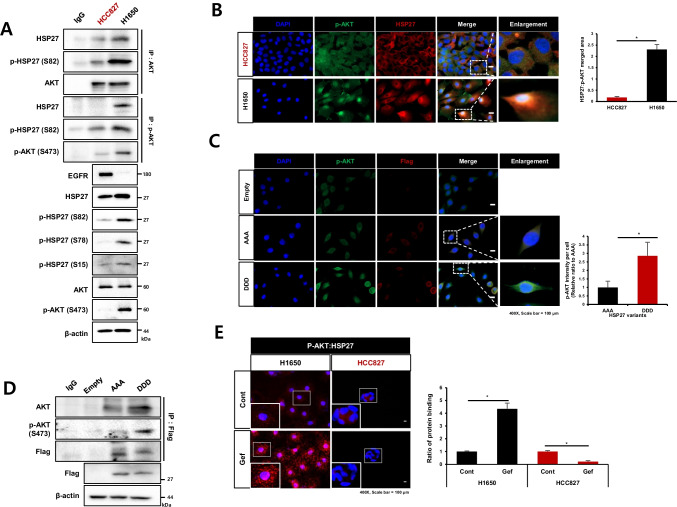
HSP27 potentiates AKT activation and stability by direct binding and induces gefitinib resistance. (**A**) Comparison of the basal protein expression levels between Gef-sensitive cells (HCC827) and Gef-resistant cells (H1650) as detected by Western blotting and direct interactions between AKT or p-AKT and HSP27 or p-HSP27 confirmed using immunoprecipitation. (**B**) Differences in colocalization between pAKT and HSP27 in HCC827 and H1650 cells using immunofluorescence. The merged area intensity of HSP27 and pAKT was quantified using ImageJ software. Student’s t-test, *p < *0.05*. (**C**) Changes in pAKT fluorescence intensity according to Flag-tagged HSP27 AAA (HSP27 phosphorylation-defective mutant at Ser15, Ser78, and Ser82) or DDD (HSP27 phosphorylation-mimicking mutant at Ser15, Ser78, and Ser82) transfection were assessed in PC9 cells. The intensity of pAKT per cell was quantified using ImageJ software. Student’s t-test, *p < *0.05*. (**D**) Confirmation of direct protein binding of AKT or pAKT and Flag-tagged HSP27 AAA or DDD in whole cell lysates of H1650 cells. After transfection of Flag-tagged HSP27 AAA or DDD, protein interactions of Flag with AKT or pAKT were analyzed by immunoprecipitation. (**E**) PLA of HSP27:pAKT and HSP27:EGFR in H1650 or HCC827 cells after treatment with 100 nM Gef for 6 h and interaction detection (red dots). Quantification of red dots was performed using ImageJ. Graphs represent the mean and S.D. of three independent experiments. Student’s t-test, *p < *0.05*

To elucidate the mechanisms involved in the relationship between HSP27 and AKT, EGFR mut-Gef-sensitive cells and EGFR-resistant cells were compared. We found that the expression of HSP27 accompanied with its phosphorylated forms at Ser15, Ser78 and Ser82 was higher in Gef-resistant cells (NCI-H1650, PC9GR) than in Gef-sensitive cells (HCC827, PC9). The expression of pAKT was also higher in NCI-H1650 and PC9GR cells than in HCC827 and PC9 cells respectively. When the binding activity between pHSP27 and pAKT was examined, a stronger binding activity was detected in Gef-resistant cells (Fig. 2A and Supplementary Fig. S2A). Immunofluorescence data revealed that HSP27 usually localized in the cytosol of Gef-sensitive HCC827 cells. In Gef-resistant NCI-H1650 cells, however, increased nuclear translocation of HSP27 was observed and to be accompanied with increased nuclear-pAKT (Fig. 2B). Similar results of stronger nuclear translocation of pAKT, as well as increased colocalization of pAKT and HSP27, were also found in PC9GR cells compared to PC9 cells (Supplementary Fig. S2B). Indeed, cellular fractionation data also indicated that Gef-resistant NCI-H1650 cells showed greater nuclear translocation of pAKT and pHSP27 than Gef-sensitive HCC827 cells (Supplementary Fig. S2C), which was affected by a higher activation of p38-MK2 pathways (Supplementary Fig. S2D). To determine whether pHSP27 is involved in the direct interaction with pAKT, phospho-defective (AAA) and mimicking (DDD) mutants of HSP27 at Ser15, Ser78 and Ser82 were prepared. Colocalization between HSP27 and pAKT in PC9 cells was stronger in DDD than in AAA mutant cells (Fig. 2C), and the binding activity of pAKT was also greater in DDD than AAA mutant cells when detected using a whole lysate of Gef-resistant cells, NCI-H1650 (Fig. 2D). Also, Gef treatment increased pHSP27 at Ser15, Ser78 and Ser82 in NCI-H1650 cells (Supplementary Fig. S2E) and cycloheximide (CHX) treatment data revealed that DDD had a longer half-life than AAA (Supplementary Fig. S2F). Using a proximity ligation assay (PLA), which can detect protein–protein binding in situ at a single-molecular resolution, endogenous interactions and localized protein interaction complexes between pAKT and HSP27 after Gef treatment were examined. A PLA for HSP27:pAKT produced abundant red puncta in Gef-treated NCI-H1650 cells, while HCC827 cells produced few red puncta, suggesting a stronger HSP27:pAKT interaction in Gef-resistant cells after Gef treatment (Fig. 2E). Immunoprecipitation data also showed more interaction between HSP27-pAKT than HSP27-EGFR in NCI-H1650 cells, while there was more interaction between HSP27-EGFR in HCC827 cells. Moreover, Gef treatment could have inhibited the HSP27-EGFR interaction, although Gef at the same concentration could not inhibit the HSP27-pAKT interaction (Supplementary Fig. S2G).

### Strong binding activity of HSP27-pAKT is involved in gefitinib resistance

**Fig. 3:**
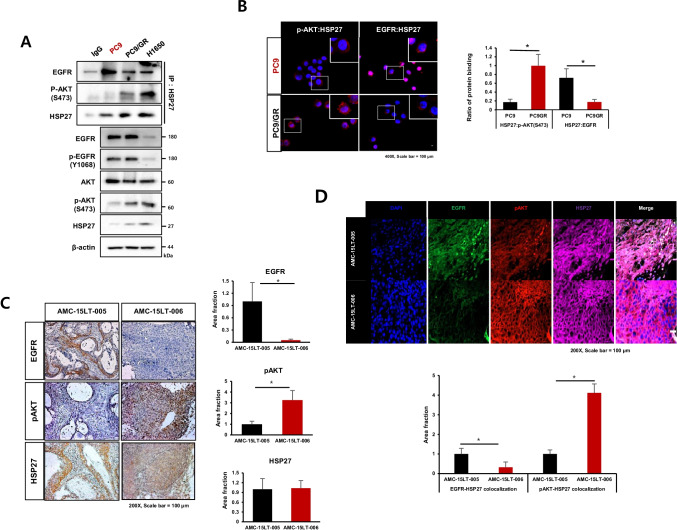

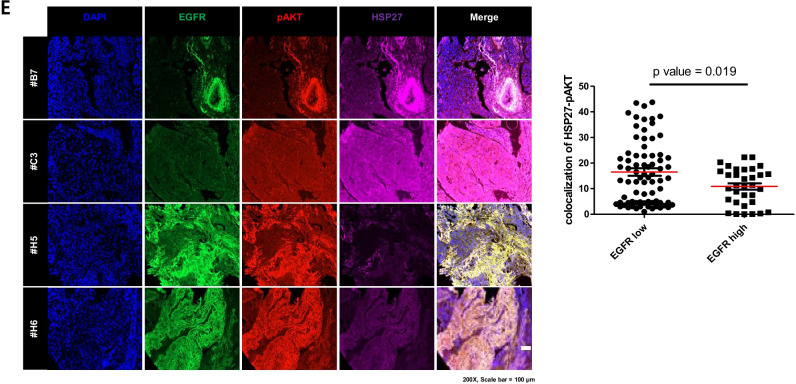
Strong HSP27-pAKT binding in gefitinib-resistant cells. (**A**) Comparison of the basal protein expression or direct interaction between Gef-sensitive cells (PC9) and Gef-resistant cells, H1650 and PC9GR, assessed by immunoprecipitation. (**B**) Proximity ligation assay of HSP27:pAKT and HSP27:EGFR in PC9 and PC9GR cells and detected interactions (red dots). Quantification of red dots was performed using ImageJ. Graphs represent the mean and S.D. of three independent experiments. Student’s t-test, *p < *0.05*. (**C**) Representative immunohistochemistry (IHC) images of EGFR, pAKT and HSP27 in PDX tumor tissues from lung cancer organoids. IHC scores were calculated using ImageJ software. Student’s t-test, *p < *0.05*. (**D**) Representative immunofluorescence images of EGFR, pAKT and HSP27 in PDX tumor tissues from lung cancer organoids. Quantification was performed on the merged intensity of HSP27 and EGFR or HSP27 and pAKT and conducted using ImageJ. 200 × magnification, Student’s t-test, * *p* < *0.05*. (**E**) Immunofluorescence for EGFR, pAKT and HSP27 using a human adenocarcinoma cancer tissue microarray. Correlation of HSP27 and pAKT merged areas and EGFR expression on each slide, quantified using ImageJ software. 200 × magnification. Student’s t-test

Since HSP27 has been reported to interact with EGFR [2], and HSP27-pAKT binding was observed in Gef-resistant cells, we compared the binding activity between HSP27-EGFR and HSP27-pAKT. We found that Gef-sensitive PC9 cells showed more interaction between HSP27-EGFR than HSP27-pAKT, whereas Gef-resistant NCI-H1650 cells showed more interaction between HSP27-pAKT than HSP27-EGFR. More HSP27-EGFR binding in PC9 cells changed to more HSP27-pAKT binding in PC9GR cells (Fig. 3A). A PLA assay using PC9 and PC9GR cells indicated more red puncta HSP27:EGFR in PC9 cells, but more red puncta HSP27:pAKT in PC9GR cells (Fig. 3B). A next set of experiments was aimed at elucidating whether HSP27-EGFR binding is predominantly induced in Gef-sensitive cells with a high expression of EGFR, which results in less binding activity between HSP27-pAKT. Transfection of NCI-H1650 cells with siAKT suggested that HSP27 interacts more with EGFR in AKT-deficient condition, and that PC9 cells with siEGFR showed that HSP27 interacted more with pAKT in EGFR-deficient condition. We confirmed the interaction switch using PLA with siEGFR-treated HCC827 cells and siAKT-treated NCI-H1650 cells (Supplementary Fig. S3A and S3B). These findings were further supported using PDX model tissues of AMC-15LT-005 and AMC-15LT-006, which carry EGFR L858R mutations. Whereas AMC-15LT-005 displayed a high sensitivity to erlotinib, an EGFR-TKI targeting EGFR mutant at L858R, as with Gef, AMC-15LT-006 was resistant to erlotinib [28]. As expected, the expression level of EGFR was higher in AMC-15LT-005 than in AMC-15LT-006, while the expression level of pAKT was lower in AMC-15LT-005 than in AMC-15LT-006. Conversely, the expression level of EGFR in AMC-15LT-006 was relatively low, while the expression level of pAKT was relatively high. No significant difference was observed in the expression of HSP27 between AMC-15LT-005 and AMC-15LT-006 (Fig. 3C). Co-localization of EGFR and HSP27 in AMC-15LT-005 was more abundantly present, while co-localization of pAKT and HSP27 was more frequent in AMC-15LT-006 (Fig. 3D). When human lung cancer tissue slides from 100 patient specimens were examined, similar expression patterns were observed (Fig. 3E). These data suggest that strong binding of HSP27-pAKT is related to EGFR TKI resistance.

### Inhibition of HSP27 reduces the stability of EGFR and pAKT

**Fig. 4:**
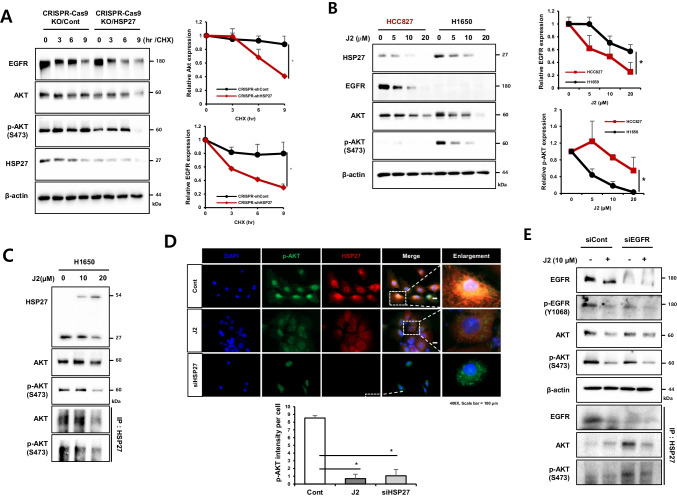
Inhibition of HSP27 attenuates EGFR and AKT stability. (**A**) Western blot analysis of CRISPR-Cas9-Control (Cont) and CRISPRT-Cas9-KO-HSP27 H1650 cells after cycloheximide (CHX) treatment at the indicated time points. Band densities are expressed as the fold changes relative to the Cont. The error bars represent S.D. Student’s t-test, *p < *0.05*. (**B**) The indicated cells were treated with J2 at different concentrations for 24 h, after which cell lysates were subjected to Western blot analysis. Band densities are expressed as fold change relative to that of 0 µM. The error bars represent S.D. Student’s t-test, *p < *0.05*. (**C**) Direct protein interactions between HSP27 and AKT (or phosphorylated form) were confirmed using immunoprecipitation in H1650 cells after treatment with J2. (**D**) Expression differences of pAKT and HSP27 after J2 treatment or HSP27 knockdown detected using immunofluorescence. The intensity of pAKT per cell was quantified using ImageJ software. (**E**) Transfection of siCont and siEGFR in PC9 according to J2 (24 h) treatment confirmed using immunoprecipitation

To investigate whether HSP27 affects EGFR or pAKT expression, we inhibited HSP27 in Gef-resistant or -sensitive cells. HSP27 depletion using a CRISP/CAS9 system decreased the expression of AKT and pAKT, as well as that of EGFR (Fig. 4A). We found that J2 inhibited HSP27 function by altering dimerization regardless of EGFR mutations (Supplementary Fig. S4A). J2 treatment dose-dependently decreased EGFR and pAKT. In particular, we found that EGFR was decreased in Gef-sensitive cells (HCC827), and that pAKT was decreased in Gef-resistant cells (H1650) (Fig. 4B). To assess whether AKT activation was involved in Gef resistance, cells were treated with AZD5363, an AKT inhibitor. The activation of AKT by Gef was inhibited, and sensitization to Gef was found in NCI-H1650 cells (Supplementary Fig. S4B). To examine whether HSP27 inhibition affected the binding activity between HSP27 and AKT or pAKT, NCI-H1650 cells were treated with J2, which inhibited binding activity between two the molecules with decreased expression of AKT and pAKT (Fig. 4C). NCI-H1650 cells with siHSP27 or treatment with J2 decreased merged areas of pAKT and HSP27 (Fig. 4D). In addition, Gef-sensitive cells were treated with siEGFR to produce changes in the expression of EGFR and pAKT by suppressing HSP27. In PC9 cells treated with siEGFR, decreased binding activity occurred between HSP27 and EGFR, but increased binding activity occurred between HSP27 and pAKT. Moreover, J2 inhibited HSP27-pAKT interaction, as well as HSP27-EGFR binding (Fig. 4E). These data suggest that inhibition of HSP27 overcomes Gef resistance by reducing the stability of EGFR or pAKT.

### Inhibition of HSP27 sensitizes NSCLC cells to gefitinib by blocking AKT activation

**Fig. 5:**
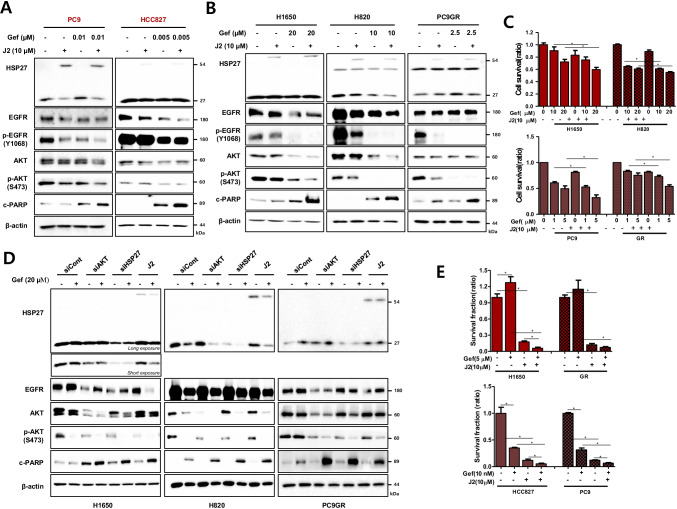
HSP27 inhibition sensitizes gefitinib-resistant NSCLC cells. (**A**) Gef-sensitive cells (PC9 and HCC827) were co-treated with J2 and Gef at 24 h, after which cell lysates were assessed by Western blot analysis. (**B**) Effect of co-treatment with J2 and Gef assessed in different cell lines after 24 h incubation. (**C**) Cell survival determined by MTT assay at 24 h of treatment. Results represent the mean and S.D. of three independent experiments. Student’s t-test, *p < *0.05*. (**D**) The indicated cells were transfected with siAKT, siHSP27 or J2 and treated with Gef (24 h), after which cell lysates were subjected to Western blot analysis. (**E**) Colony formation assay performed in the indicated cell line after treatment with Gef and J2 (long-term proliferation). Results represent the mean and S.D. of three independent experiments. Student’s t-test, *p < *0.05*

Gef-sensitive EGFR mut cells such as HCC827 and PC9 cells showed a dramatic decrease in EGFR after treatment with very low doses of Gef (5 and 10 nM). Even low doses of Gef sufficiently induced cleaved PARP and pAKT inhibition, whereas J2 showed more sensitizing effects (Fig. 5A). However, Gef-resistant NCI-H1650 and NCI-H820 cells required very high doses of Gef (10 and 20 μM) to induce cell death, which was well correlated with AKT and pAKT expression. NCI-H1650 cells showed a greater resistance to Gef than NCI-H820 cells, even though NCI-H820 cells carried an additional T790M mutation (Supplementary Fig. S5A). Moreover, Gef-resistant cells had synergistic sensitization effects with the combination of Gef with J2, as demonstrated by increased cleaved PARP and cell death rates. Although NCI-H820 cells exhibited a greater sensitivity to treatment with Gef alone than NCI-H1650 cells, sensitizing effects in combination with J2 were stronger in NCI-H1650 than NCI-H820 cells and were well correlated with pAKT expression. In addition, NCI-H1650, NCI-H820 and PC9GR cells exhibited an increased sensitivity to Gef when J2 was combined (Fig. 5B and C). Similar to intrinsic resistant NCI-H1650 and NCI-H820 cells, knockdown of HSP27 and AKT or J2 treatment in PC9GR cells resulted in a dramatic increase in sensitization when combined with Gef (Fig. 5D). Furthermore, a sensitization effect of Gef in combination with J2 was induced when long-term proliferation effects were examined using colony forming assays in Gef-sensitive and -resistant cells (Fig. 5E). Moreover, the J2 combination showed sensitization even in EGFR wt NCI-H460 and A549 cells, i.e., sensitization in the combination of Gef with J2 was more dramatic in A549 cells with a higher expression of HSP27 and pAKT than in NCI-H460 cells and a dramatic decrease of pAKT occurred in A549 cells treated with the combination of Gef and J2 (Supplementary Fig. S5B and S5C). Similarly, knockdown of AKT and HSP27 or J2 treatment in combination with Gef in A549 cells led to increased cleaved PARP expression and reduced pAKT expression (Supplementary Fig. S5D). In addition, combination indices (CIs) indicated that J2 showed a synergistic sensitization effect in combination with Gef in all the cell lines tested, regardless of EGFR mutations (Table 2).

**Table 2:** CI values in combination of gefitinib and J2 in NSCLC cell lines

Cell lines	Gef (µM)	Combination Index (CI)
NCI-H460	5	0.3456
	10	0.3205
A549	10	0.6079
	20	0.7548
HCC827	0.001	0.3216
	0.005	0.1748
PC9	0.001	0.279
	0.005	0.5954
NCI-H1650	10	0.6513
	20	0.5268
NCI-H820	5	0.5298
	10	0.6931
NCI-H1975	10	0.7066
	20	0.4865
PC9GR	1	0.4959
	5	0.2709

Each graph is the representative combination indices (CIs) of Gef in combination with J2, 10 µM: CI < 1 indicates synergy, CI = 1 is summation, and CI > 1 indicates antagonism. CI values were calculated from the data of percent inhibition of cell death by MTT assay. Each CI value was analyzed by CompuSyn software

### The HSP27-pAKT axis is more strongly involved in gefitinib resistance than secondary EGFR mutations such as T790M

**Fig. 6:**
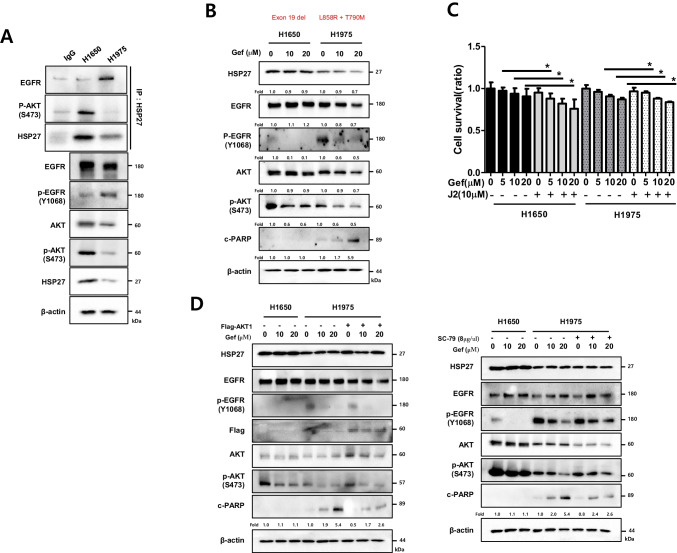
HSP27-pAKT binding is more important for gefitinib resistance than is the EGFR mutation T790M. (**A**) Comparison of the basal protein expression and direct interaction with HSP27 between EGFR and pAKT detected by immunoprecipitation in H1650 and H1975 cells. (**B**) The indicated cells were treated with Gef at different concentrations for 24 h, after which cell lysates were subjected to Western blot analysis. (**C**) Cell survival determined by MTT assay at 24 h of treatment. Results represent the mean and S.D. of three independent experiments. Student’s t-test, *p < *0.05*. (**D**) The indicated cells were treated with Gef (8 h) after transfection with empty vector or Flag-AKT constructs (left). At 30 min before treatment, SC-79 (8 µg/ul) (right) and Western blotting were performed. Fold represents band density relative to control

NCI-H820 cells showed greater sensitivity to Gef than NCI-H1650 despite the presence of additional secondary mutations such as T790M, which might be caused by the lower expression of pAKT. To confirm whether pAKT expression is more important than the T790M mutation in Gef resistance, NCI-H1975 cells, which carry both L858R and T790M mutations, were compared with NCI-H1650 cells. We found that NCI-H1650 cells showed a higher expression of pAKT as well as a higher expression of HSP27 than NCI-H1975 cells, whereas EGFR activation was higher in NCI-H1975 cells than in NCI-H1650 cells. Binding activity between HSP27 and EGFR was higher in NCI-H1975 cells, whereas the interaction of HSP27 with pAKT was greater in NCI-H1650 cells (Fig. 6A). Gef sensitivity was higher in NCI-H1975 cells than in NCI-N1650 cells (Fig. 6B). Moreover, when J2 was combined with Gef, a greater combined effect was observed in NCI-H1650 cells than in NCI-H1975 cells (Fig. 6C). To clarify whether reduced pAKT expression in NCI-H1975 cells was responsible for Gef sensitivity, AKT wt was transfected into NCI-1975 cells, after which Gef sensitivity was examined. It was found that AKT overexpression in NCI-H1975 cells resulted in a lower expression of PARP cleavage without alteration of EGFR activation (Fig. 6D, left). In addition, treatment of NCI-H1975 cells with SC-79, an AKT activator [25], decreased Gef sensitivity compared with untreated control cells (Fig. 6D, right).

### Overcoming gefitinib resistance by inhibition of HSP27 in NSCLC xenograft mouse systems and positive correlation between the expression of HSP27 and pAKT in human lung cancer tissues

**Fig. 7:**
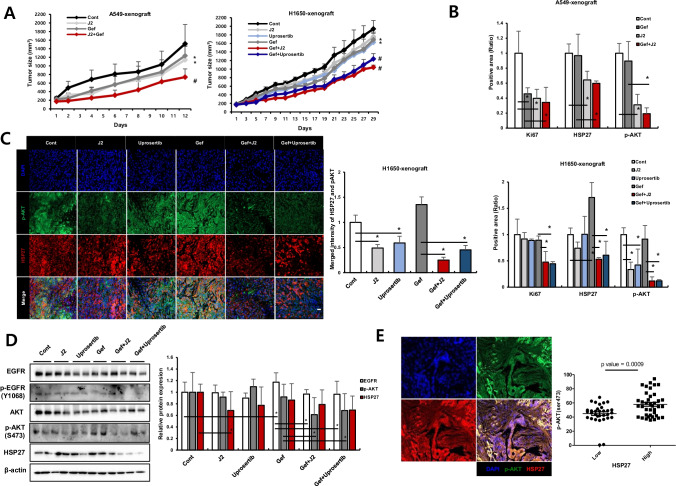

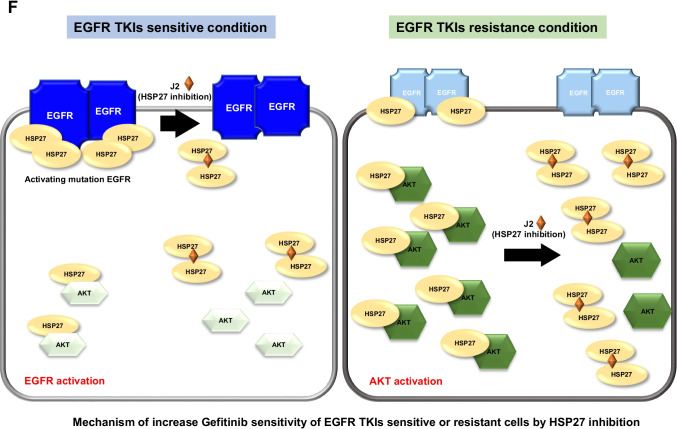
HSP27 inhibition overcomes gefitinib-mediated resistance in a NSCLC xenograft model. (A) *In vivo* tumor growth after treatment with J2 was assessed using A549 and NCI-H1650 (H1650) xenograft mouse models (n = 5 per group, i.p., J2 was administered every other day, Gef and Uprosertib were applied once every 3 days). Tumor volumes were calculated using the equation (length × width^2^)/2 (mean ± S.E.M). Student’s t-test, *p < *0.05*. (**B**) Representative immunohistochemistry (IHC) images of Ki67, HSP27 and pAKT from the tumors described above. IHC scores were calculated using ImageJ software. Student’s t-test, *p < *0.05*. (**C**) Representative immunofluorescence images of pAKT and HSP27 in tumor tissue from H1650 xenograft mice treated with each of the indicated drugs. Quantification was performed on the merged intensity of HSP27 and pAKT and conducted using ImageJ. 200 × magnification, Student’s t-test, * *p* < *0.05*. (**D**) Representative blots of protein expression in tumor tissues from H1650 xenograft mice treated with each of the indicated drugs using immunoblotting (n = 2). Protein levels were quantified using Image J software. The data are expressed as fold change relative to the control (n = 5). Student’s t-test, * *p* < *0.05*. (**E**) Immunofluorescence for HSP27 and pAKT in a human adenocarcinoma cancer tissue microarray performed using fluorescence-conjugated antibodies. Photographs of four representative cancer tissues are presented. Quantification of HSP27-positive and pAKT-positive areas in each slide were performed using ImageJ software. (**F**) Graphical summary of HSP27-mediated Gef resistance and targeting the HSP27-pAKT axis by HSP27 inhibitors for overcoming Gef resistance. In NSCLC cells with Gef-sensitive EGFR mutations, HSP27 (or pHSP27) preferentially binds to overexpressed EGFR and regulates the activity and stability of the EGFR. However, in Gef-resistant NSCLC cells, regardless of EGFR mutations, high pAKT and HSP27 (pHSP27) expression was observed. In this case, HSP27 preferentially binds to AKT (or pAKT) and increases its protein stability, causing Gef resistance. Therefore, functional inhibition of HSP27 using J2, a small molecule inducing cross-linking of HSP27, can induce sensitization to Gef and suggests a new strategy to overcome Gef resistance by inhibition of AKT pathways

*In vivo* data using mice grafted with A549 (EGFR wt/Gef-resistant) and NCI-H1650 (EGFR mut/Gef-resistant) cells indicated that J2 treatment led to sensitization in combination with Gef. The sensitizing effects of J2 with Gef were similar to those of the combination treatment of Gef and Uprosertib, a small-molecule AKT inhibitor (Fig, 7A). In addition, Ki67-positive cells in tumor tissues correlated well with the sensitizing effects of J2 or Uprosertib in combination with Gef. The weaker inhibition of tumor volume for the combined treatment with J2 or Uprosertib relative to that of Ki67-positive cells may have occurred because J2 or Uprosertib did not affect immune cells like CD3 positive cells, which account for a part of the tumor volume, but only affected cancer cells (Supplementary Fig. S6A). Immunohistochemistry and immunofluorescence data for pAKT and HSP27 revealed that J2 inhibited pAKT expression and colocalization between HSP27 and pAKT (Fig. 7B and C and Supplementary Fig. S6B). Moreover, immunoblotting using xenograft tumor tissues suggested that pAKT and HSP27 were inhibited by combined treatment of J2 (Fig. 7D). When human lung cancer tissue slides of 75 patient specimens were examined, similar expression patterns were observed (Fig. 7E and Supplementary Fig. S6C), suggesting a positive correlation between expression of HSP27 and pAKT. Our findings suggest that inhibition of HSP27 overcomes Gef resistance regardless of EGFR mutation.

## Discussion

Our findings suggest that AKT activation plays a major role in the resistance of first-generation EGFR-TKIs such as Gef, and that HSP27 overexpression is involved in AKT activation by direct interaction, especially of phosphorylated forms. Moreover, it was confirmed that HSP27-mediated AKT activation is more strongly involved in Gef resistance than are secondary mutations of EGFR such as T790M, which is known to induce resistance to first-generation EGFR-TKIs. Large-scale cohort analysis revealed that expression of HSP27 and mutant EGFR were closely related to the prognosis of lung cancer patients. Indeed, EGFR mut cells showed a higher expression of HSP27, and Gef resistance was not correlated with the status of pEGFR but, instead, with pAKT in both EGFR wt and EGFR mut cells. In other downstream pathways of EGFR, such as pSTAT and pERK, no correlation with Gef resistance was observed. Recent studies reported that the expression of HSP27 is increased and confers resistance in oncogene-addicted cells upon tyrosine kinase inhibition [29, 30], but the exact mechanism remained unclear. To assess whether HSP27-mediated pAKT is involved in Gef resistance, cell lines were treated with various doses of Gef, after which we found that pEGFR responded in a dose-dependent manner in all cell lines tested, regardless of EGFR wt and mut status. However, Gef resistance correlated well with pAKT. Gef-sensitive cell lines showed decreased pAKT and AKT downstream pathway activities depending on Gef concentration, while Gef-resistant cell lines did not show any decrease in pAKT or its downstream pathways, which occurred in both EGFR wt and mut cells. Moreover, clinic-genomic data revealed that HSP27 was more strongly involved in cancer prognosis than EGFR or AKT alone, and that overexpression of HSP27 potentiated negative effects in NSCLC patients.

HSP27 serves as an upstream factor of AKT and interacts directly with AKT to control apoptosis, p38 kinase and MK2, and HSP27 forms a signaling complex with AKT [17–19]. Phosphorylation of AKT is maintained or prolonged after HSP27 overexpression, which protects AKT from dephosphorylation [31]. Therefore, HSP27 is necessary for AKT activation. Our data revealed that Gef treatment increased HSP27 expression and phosphorylation. Phosphorylated HSP27 interacted with AKT, and this interaction was strengthened in pAKT, which facilitated the activation of AKT. HSP27-regulated AKT activation, and pHSP27 can more strongly chaperone AKT and stabilize it to control cell survival, which may involve Gef resistance. Increased expression of pHSP27 was mediated by increased activation of pp38 and pMK2, and phosphorylation of HSP27 increased its stability. It was also confirmed that resistant PC9GR cells, which were prepared by treating PC9 cells repeatedly with Gef, exhibited increased expression levels of HSP27 and pHSP27, as well as pp38 and pMK2. Moreover, our results indicate that increased expression of pHSP27 potentiated nuclear AKT translocation, which might be responsible for Gef resistance. Constitutively activated AKT accumulated in the nucleus and regulated oncogenic proteins. Nuclear translocation of AKT after phosphorylation has been reported to involve cellular proliferation and cell survival [32, 33].

HSP27 in Gef-sensitive EGFR mut cells interacted with EGFR rather than pAKT. Gef-sensitive EGFR mut cells showed a higher expression of EGFR and, therefore, HSP27 primarily interacted with EGFR. While Gef-sensitive EGFR mut cells showed a lower expression of pAKT, in Gef-resistant EGFR wt and mut cells HSP27 interacted more with the more highly expressed pAKT, which may be mediated by less interaction with EGFR. Moreover, AKT knockdown potentiated the HSP27-EGFR interaction instead of that of HSP27-pAKT with Gef sensitization. On the contrary, EGFR knockdown in Gef-sensitive cells potentiated the HSP27-pAKT interaction. Although we do not exactly know why HSP27 interacts differently with EGFR and AKT in Gef-sensitive cells or Gef-resistant cells, one of the mechanisms may be that the amount of EGFR is high enough in Gef-sensitive cells, so HSP27 can sufficiently interact with EGFR. However, in resistant cells, EGFR is not sufficient to interact with HSP27 and other signaling pathways such as AKT are activated. Therefore, the interaction between HSP27 and AKT (pAKT) may occur preferentially. Indeed, AKT activation has been reported to be involved in the activation of other receptor TKs (HER2, FGFR and MET) [11], which may more potently activate AKT and the binding activity of HSP27 with pAKT. Our data also indicate that Gef-sensitive cell lines showed a higher EGFR expression and a stronger HSP27-EGFR binding, and that Gef-resistant cell lines with a higher AKT expression and activation showed a stronger HSP27-AKT (pAKT) binding. Therefore, combination treatment of Gef with a HSP27 inhibitor like J2 may be a more attractive strategy in both Gef-sensitive and -resistant cases. Our PDX model also indicated that EGFR-TKI resistant cells show a higher pAKT expression (lower expression of EGFR) compared to EGFR-TKI sensitive cells (higher expression of EGFR), supporting the relevance to pathological situations of human NSCLC. Moreover, human NSCLC tissues also indicated that the expression levels of HSP27 and pAKT were well correlated, and that there was an inverse correlation between EGFR and pAKT in the co-localization of HSP27.

Another important finding was the involvement of pAKT in Gef resistance. Secondary mutations such as T790M have been reported to be involved in Gef resistance [2–5]. Our results indicate, however, that AKT activation including downstream pathways of AKT are more predominantly involved in Gef resistance than T790M, and that HSP27 potentiates AKT activation by direct interaction. Moreover, recent clinical data suggested a convergent role of AKT activation in acquired EGFR-TKI resistance that is associated with diverse and unpredictable upstream molecular events [11]. Osimertinib is a third-generation EGFR-TKI that is highly selective for EGFR-activating mutations as well as the EGFR T790M mutation in patients with NSCLC. Despite the documented efficacy of osimertinib in first- and second-line settings, patients inevitably develop resistance including bypass pathway activation and downstream pathway activation like AKT activation [34]. No clear-cut therapeutic options are available to date other than chemotherapy and locally ablative therapy. HSP27 inhibitors would provide an option for overcoming resistance in NSCLC.

The AKT signaling pathway has emerged as an important target in cancer therapy. Numerous AKT pathway inhibitors are being studied and are undergoing clinical trials. However, despite the multitude of clinical studies for the treatment of cancer, none of the targeted AKT inhibitors have reached phase III in clinical trials, reflecting the enormous complexity of AKT-signal-dependent malfunctions and potential adverse effects that have largely restricted the application and clinical significance of these inhibitors [35]. Our current results may provide valuable insights that can be used to develop new cancer treatment strategies for NSCLC patients, including those who are first-generation EGFR-TKI resistant, without toxicity from direct treatment of AKT inhibitors.

Recent studies have suggested the potential of inhibiting HSP27 as a therapeutic target for cancer. However, it is believed that, unlike other heat shock proteins such as HSP70 and HSP90, sHSPs such as HSP27 lack an ATP binding site, and this makes it difficult to consider HSP27 as an easy target for a small molecule inhibitor. Since antisense oligonucleotide drugs such as OGX-427 are still undergoing clinical trials, researchers should focus their efforts in this direction to investigate potential new cancer therapies. In this sense, small molecules like J2 that cross-link with HSP27 have the potential for functional inhibition of HSP27 [36]. More importantly, our data suggest that J2-mediated sensitization with Gef is a long term effect, not a transient one. Overcoming Gef resistance by HSP27 inhibitors has limitations, i.e., our *in vivo* xenograft data did not indicate a dramatic effect, although sensitizing effects of HSP27 inhibitor were observed in long-term effects like colony forming assays. This might be an effect of offsetting the difference in the actual tumor volume due to immune cell infiltration, which is common in an *in vivo* model. In fact, we observed no change associated with J2 treatment in CD3-positive cells. Another factor may be limitations of the *in vivo* drug efficacy of J2, possibly associated with limitations on the infiltration of J2 into the tumor mass. These problems remain to be resolved.

In conclusion, we found that HSP27 is frequently overexpressed in NSCLC and is associated with resistance development against Gef. Co-administration of small-molecule HSP27 inhibitors, such as J2, should be considered because they may improve cancer-targeted therapy in NSCLC by pAKT inhibition, which is a critical factor for Gef resistance and may provide an important step forward given the limitations of existing treatment options.

## Supplementary Information

Below is the link to the electronic supplementary material.

Supplementary file1 (XLSX 21 KB)

Supplementary file2 (PPTX 27064 KB)

Supplementary file3 (DOCX 26 KB)

## Data Availability

The datasets used and/or analyzed during the current study are available from the corresponding author upon reasonable request.

## Abbreviations

EGFR: Epidermal growth factor receptor
TKI: Tyrosine kinase inhibitor
NSCLC: Non-small cell lung cancer
HSP27: Heat shock protein 27
Gef: Gefitinib
TCGA: The cancer genome atlas
AKT: Protein kinase B (PKB) or serine/threonine-specific protein kinase
PI3K: Phosphoinositide 3-kinases
mTOR: Mammalian target of rapamycin
MAPK: Mitogen-activated protein kinase
JAK: Janus kinase
STAT: Signal transducer and activator of transcription
MK2: MAP kinase-activated protein kinase 2
PRAS40: Proline-rich AKT substrate of 40 kDa
LCO: Lung cancer organoid
