# Supplementary material for: Activation of the HSP27-AKT axis contributes to gefitinib resistance in non-small cell lung cancer cells independent of EGFR mutations
Source: Cell Oncol (Dordr). 2022 Aug 5;45(5):913–30. doi: 10.1007/s13402-022-00696-3 (PMC9579113; doi:10.1007/s13402-022-00696-3)
Supplement: Supplementary file 2 — Supplementary file2 (PPTX 27064 KB) [file 13402_2022_696_MOESM2_ESM.pptx]

## Slide 1
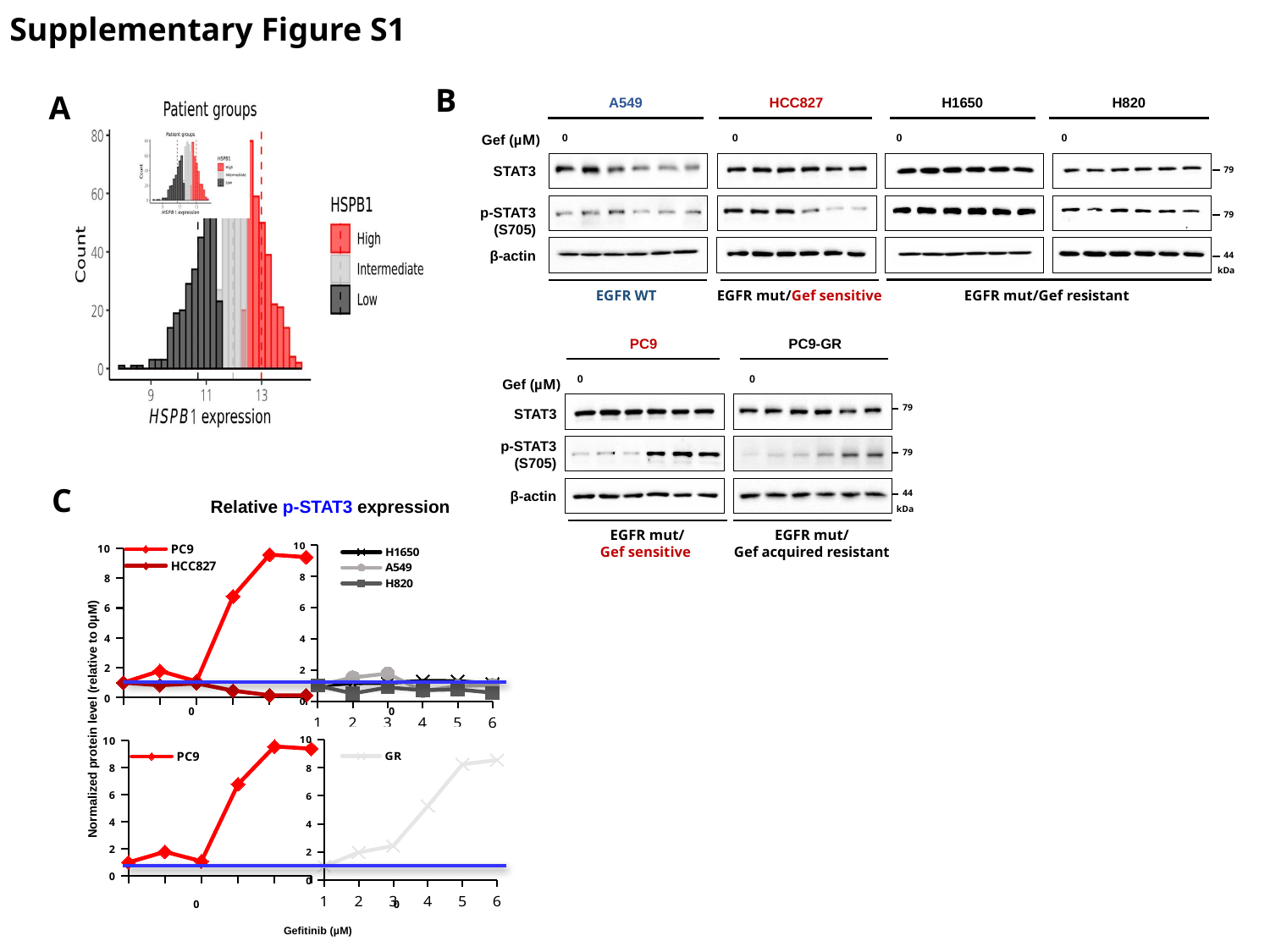

Supplementary Figure S1
B
A
A549
HCC827
H1650
H820
 Gef (µM)
STAT3
p-STAT3
(S705)
β-actin
EGFR mut/Gef sensitive
EGFR mut/Gef resistant
EGFR WT
79
79
44
kDa
PC9
PC9-GR
 Gef (µM)
79
STAT3
p-STAT3
(S705)
79
β-actin
44
kDa
EGFR mut/
Gef acquired resistant
EGFR mut/
Gef sensitive
C
Relative p-STAT3 expression
Normalized protein level (relative to 0µM)
Gefitinib (µM)

## Slide 2
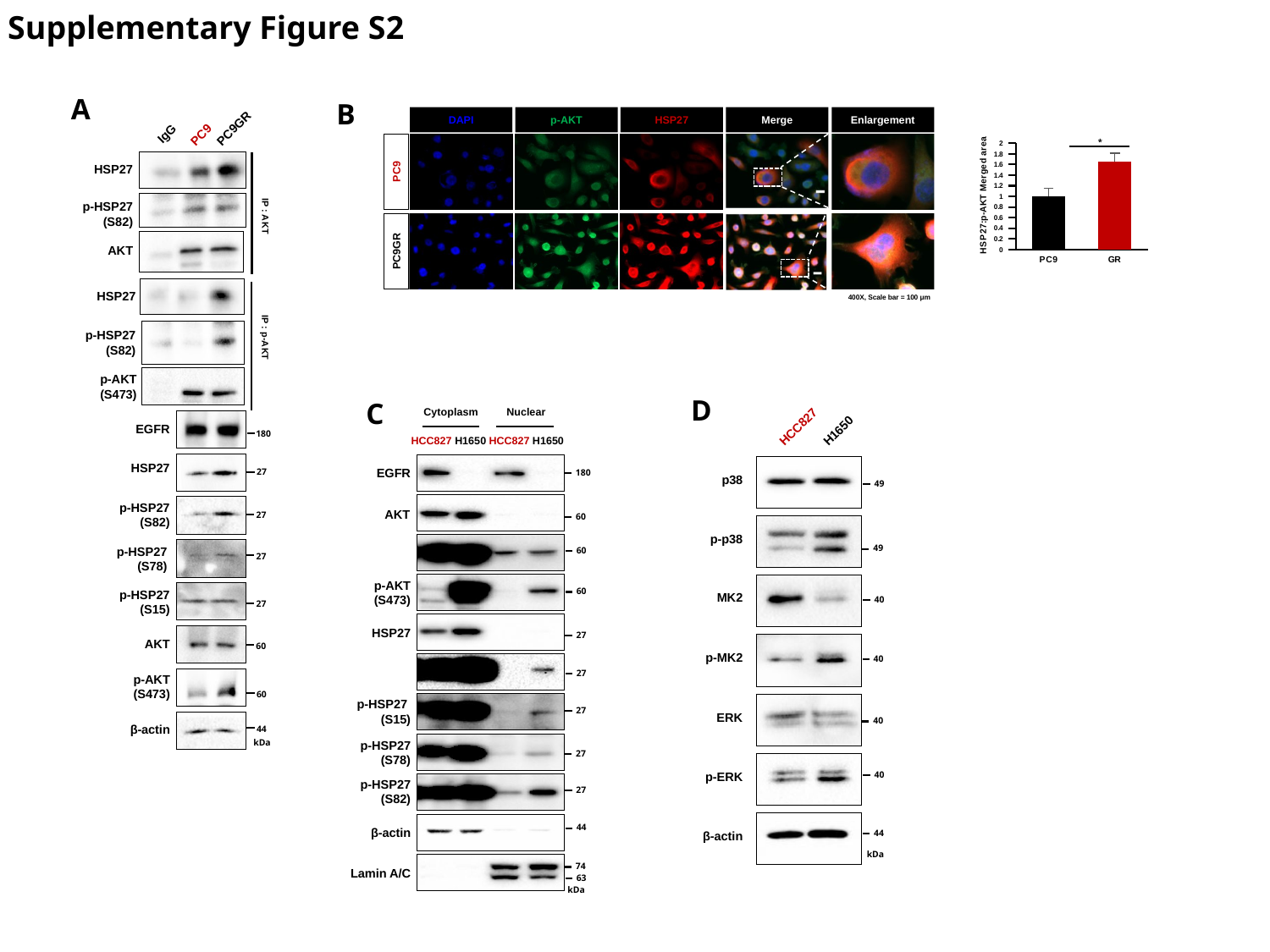

Supplementary Figure S2
A
B
DAPI
p-AKT
HSP27
Merge
Enlargement
PC9
PC9GR
400X, Scale bar = 100 μm
IgG
PC9GR
PC9
### Chart
| Category | HSP27:p-AKT Merged area |
|---|---|
| PC9 | 1.0 |
| GR | 1.6528243292917568 |*
HSP27
p-HSP27
(S82)
IP : AKT
AKT
HSP27
p-HSP27
(S82)
IP : p-AKT
p-AKT
(S473)
D
C
Cytoplasm
Nuclear
HCC827
H1650
p38
49
p-p38
49
MK2
40
p-MK2
40
ERK
40
40
p-ERK
44
β-actin
kDa
EGFR
180
HCC827 H1650 HCC827 H1650
HSP27
EGFR
27
180
p-HSP27
(S82)
AKT
27
60
p-HSP27
(S78)
60
27
Long exposure
p-AKT
(S473)
60
p-HSP27
(S15)
27
HSP27
27
AKT
60
27
p-AKT
(S473)
Long exposure
60
p-HSP27
(S15)
27
β-actin
44
kDa
p-HSP27
(S78)
27
p-HSP27
(S82)
27
44
β-actin
74
Lamin A/C
63
kDa

## Slide 3
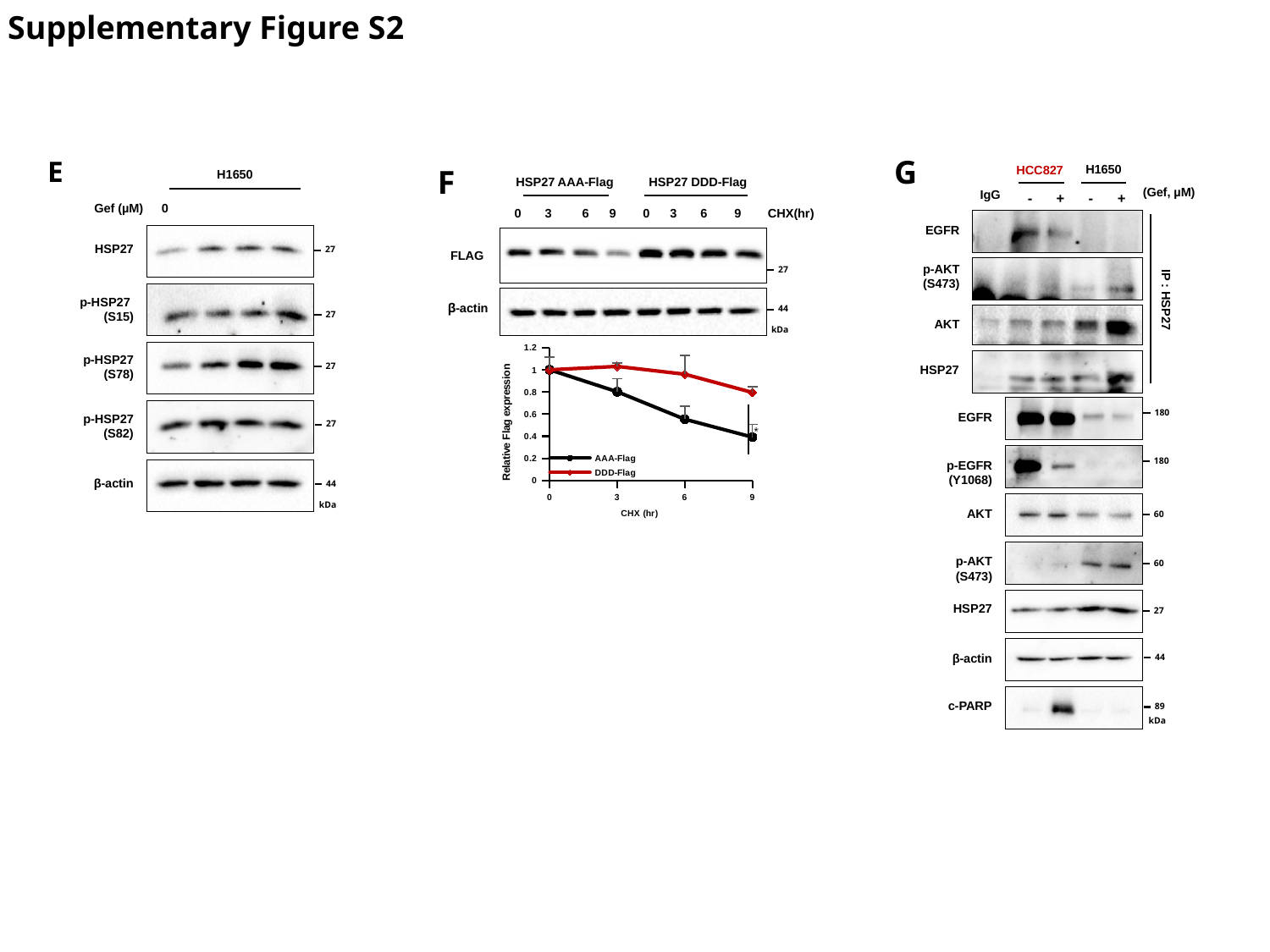

Supplementary Figure S2
G
E
H1650
HCC827
F
H1650
HSP27 DDD-Flag
HSP27 AAA-Flag
CHX(hr)
FLAG
β-actin
 0 3 6 9 0 3 6 9
27
44
kDa
IgG
 - + - +
Gef (µM)
EGFR
HSP27
27
p-AKT
(S473)
p-HSP27
(S15)
IP : HSP27
27
AKT
### Chart
| Category | AAA-Flag | DDD-Flag |
|---|---|---|
| 0 | 1.0000000965107105 | 0.9999995141267024 |
| 3 | 0.8016289886191851 | 1.0304123577499043 |
| 6 | 0.5534946738295224 | 0.9598683413960094 |
| 9 | 0.3915718341328336 | 0.795483922356181 |*
p-HSP27
(S78)
27
HSP27
180
EGFR
p-HSP27
(S82)
27
180
p-EGFR
(Y1068)
β-actin
44
kDa
AKT
60
p-AKT
(S473)
60
HSP27
27
β-actin
44
c-PARP
89
kDa

## Slide 4
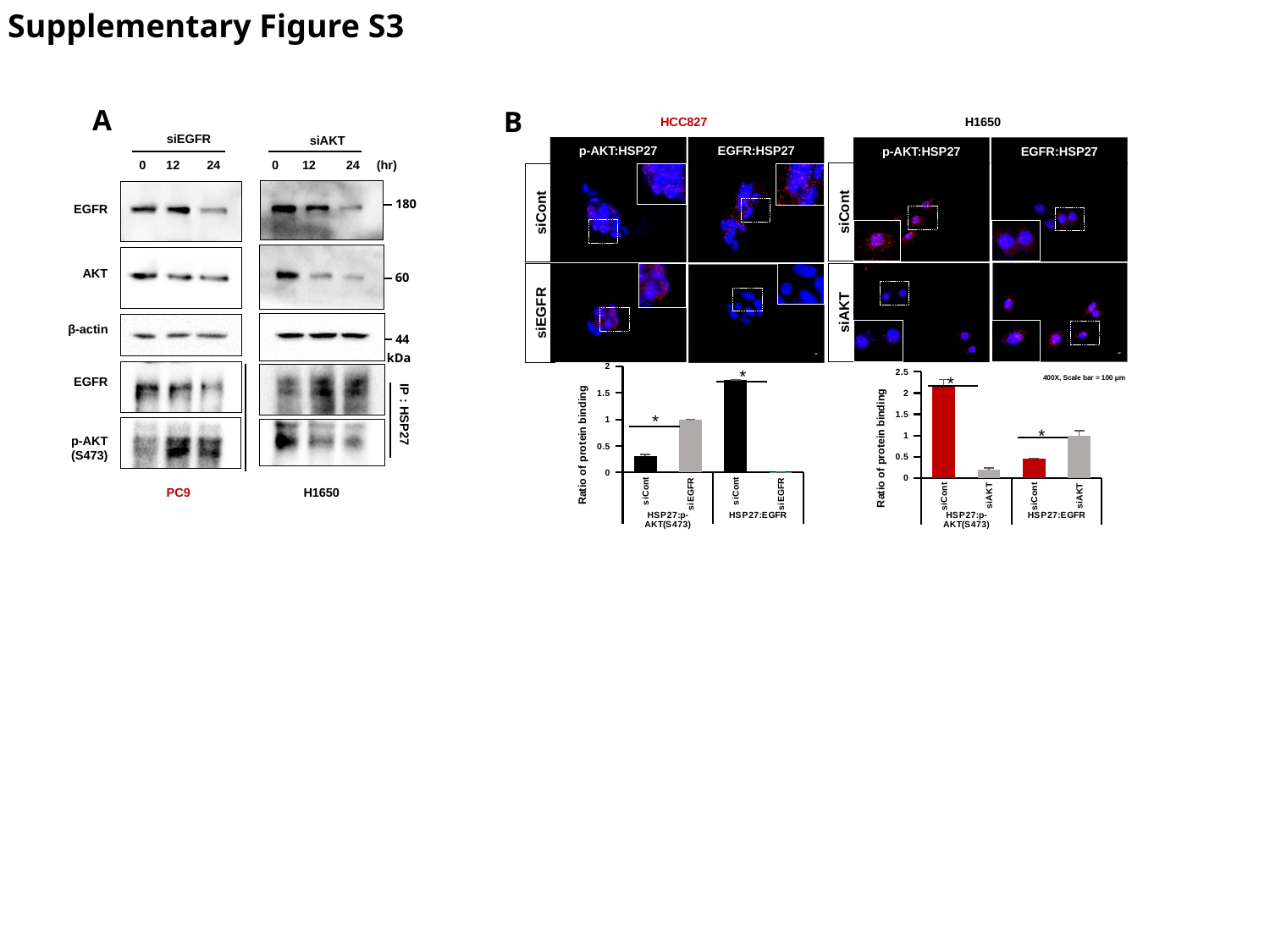

Supplementary Figure S3
A
B
HCC827
H1650
siEGFR
siAKT
0 12 24
0 12 24 (hr)
EGFR
AKT
β-actin
EGFR
p-AKT
(S473)
180
60
44
kDa
IP : HSP27
PC9
H1650
p-AKT:HSP27
EGFR:HSP27
siCont
siEGFR
p-AKT:HSP27
EGFR:HSP27
siCont
siAKT
### Chart
| Category | |
|---|---|
| siCont | 0.31000000000000005 |
| siEGFR | 1.0 |
| siCont | 1.7400000000000009 |
| siEGFR | 6.666666666666668e-05 |*
*
### Chart
| Category | |
|---|---|
| siCont | 2.1839963833634717 |
| siAKT | 0.18597857838364168 |
| siCont | 0.44345991561181436 |
| siAKT | 1.0 |*
*
400X, Scale bar = 100 μm

## Slide 5
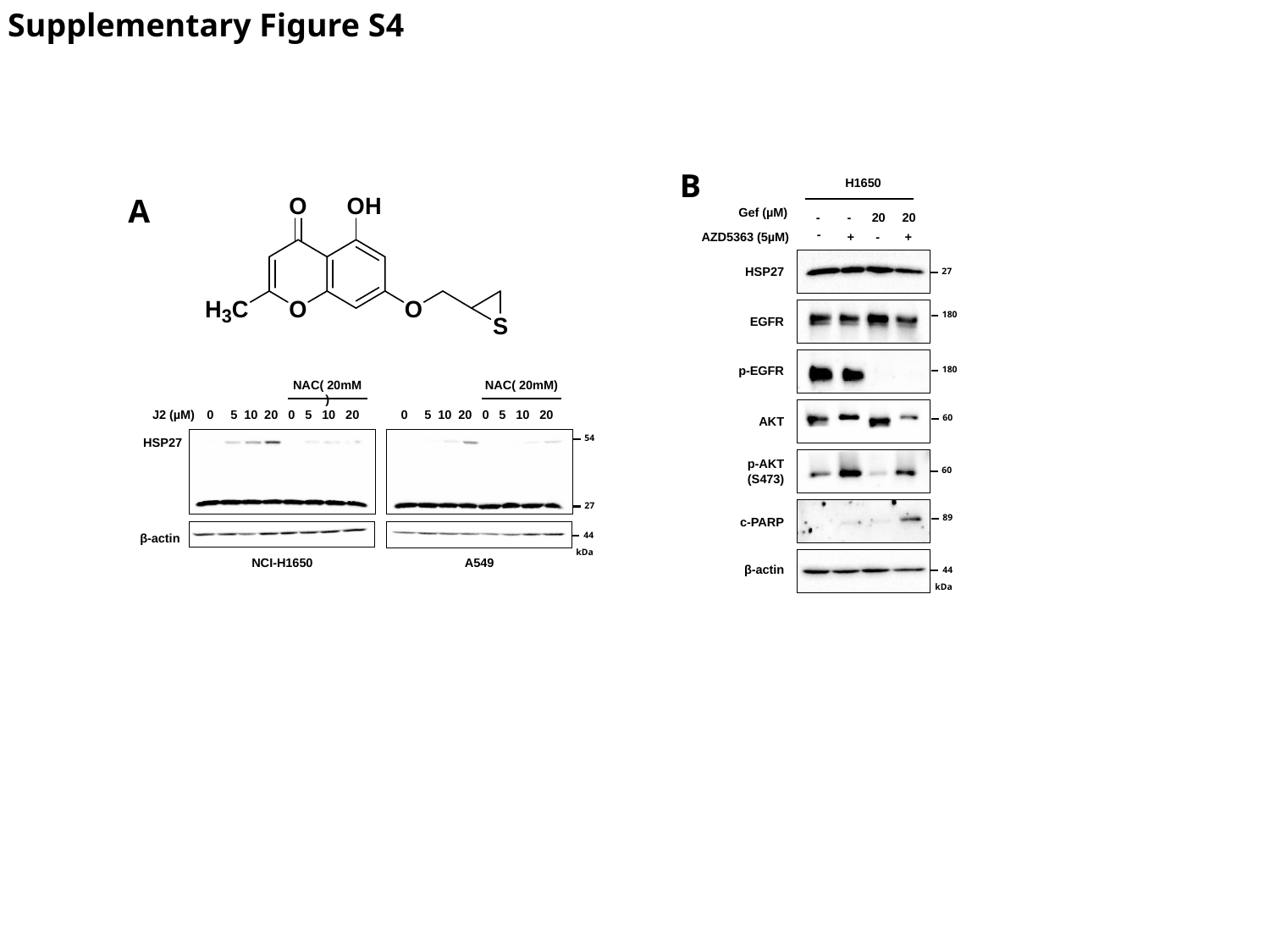

Supplementary Figure S4
B
H1650
A
Gef (µM)
-
 -
20
20
-
 +
 +
-
AZD5363 (5µM)
HSP27
27
180
EGFR
p-EGFR
180
NAC( 20mM)
NAC( 20mM)
J2 (µM)
 0 5 10 20 0 5 10 20
 0 5 10 20 0 5 10 20
NCI-H1650
A549
60
AKT
54
HSP27
p-AKT
(S473)
60
27
89
c-PARP
44
β-actin
kDa
β-actin
44
kDa

## Slide 6
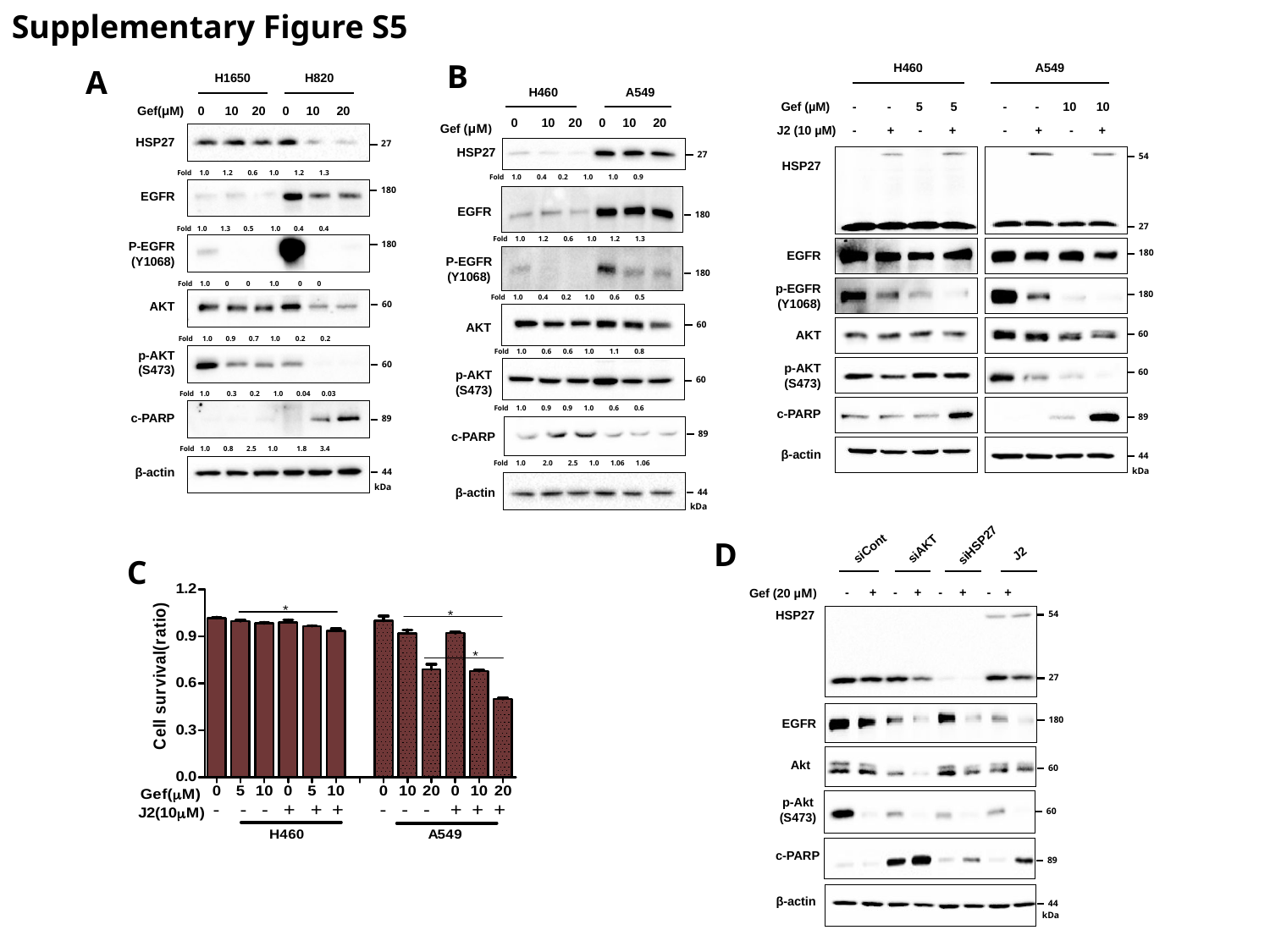

Supplementary Figure S5
B
H460
A549
Gef (µM)
-
-
 5
 5
-
-
10
10
 -
 +
 -
 +
 -
 +
 -
 +
J2 (10 µM)
54
HSP27
27
180
EGFR
p-EGFR
(Y1068)
180
AKT
60
p-AKT
(S473)
60
c-PARP
89
β-actin
44
kDa
A
H1650
H820
H460
A549
Gef(µM)
 0 10 20 0 10 20
 0 10 20 0 10 20
Gef (µM)
HSP27
27
HSP27
27
Fold 1.0 1.2 0.6 1.0 1.2 1.3
 Fold 1.0 0.4 0.2 1.0 1.0 0.9
180
EGFR
EGFR
180
Fold 1.0 1.3 0.5 1.0 0.4 0.4
Fold 1.0 1.2 0.6 1.0 1.2 1.3
P-EGFR
(Y1068)
180
P-EGFR
(Y1068)
180
Fold 1.0 0 0 1.0 0 0
 Fold 1.0 0.4 0.2 1.0 0.6 0.5
60
AKT
60
AKT
Fold 1.0 0.9 0.7 1.0 0.2 0.2
 Fold 1.0 0.6 0.6 1.0 1.1 0.8
p-AKT
(S473)
60
p-AKT
(S473)
60
Fold 1.0 0.3 0.2 1.0 0.04 0.03
 Fold 1.0 0.9 0.9 1.0 0.6 0.6
c-PARP
89
89
c-PARP
Fold 1.0 0.8 2.5 1.0 1.8 3.4
 Fold 1.0 2.0 2.5 1.0 1.06 1.06
β-actin
44
kDa
β-actin
44
kDa
D
siHSP27
siCont
siAKT
J2
C
 - + - + - + - +
 Gef (20 µM)
HSP27
54
27
180
EGFR
Akt
60
p-Akt
(S473)
60
c-PARP
89
β-actin
44
kDa

## Slide 7
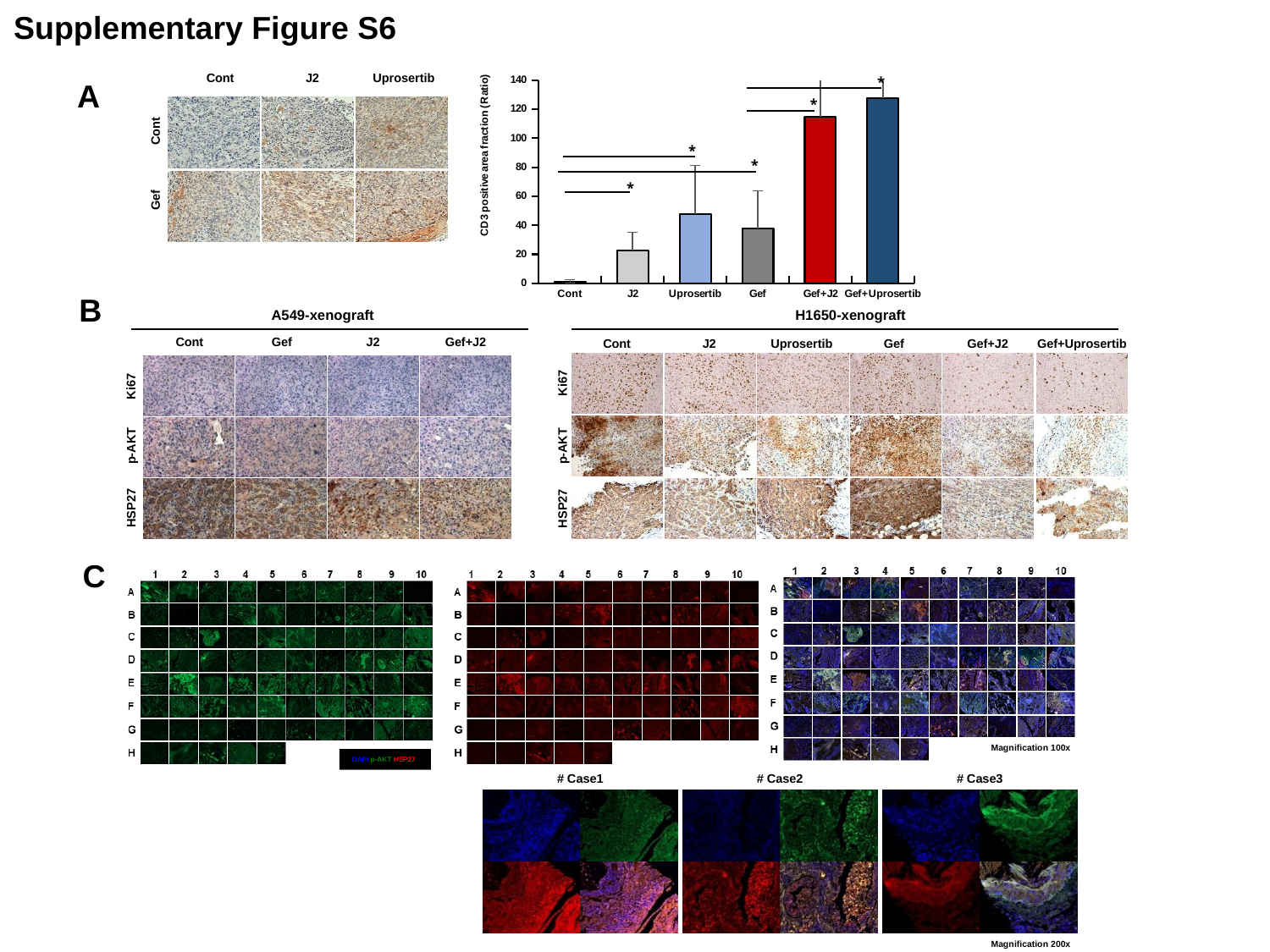

Supplementary Figure S6
Cont
J2
Uprosertib
Cont
Gef
*
### Chart
| Category | |
|---|---|
| Cont | 1.0000023992380018 |
| J2 | 22.847793540966755 |
| Uprosertib | 47.51834817262037 |
| Gef | 37.49481164782066 |
| Gef+J2 | 114.87311629826367 |
| Gef+Uprosertib | 127.4883097128352 |*
*
*
*
A
B
A549-xenograft
H1650-xenograft
Cont
Gef
J2
Gef+J2
Cont
J2
Uprosertib
Gef
Gef+J2
Gef+Uprosertib
Ki67
Ki67
p-AKT
p-AKT
HSP27
HSP27
C
Magnification 100x
DAPI/p-AKT/HSP27
# Case1
# Case2
# Case3
Magnification 200x
