# Supplementary material for: Activation of the HSP27-AKT axis contributes to gefitinib resistance in non-small cell lung cancer cells independent of EGFR mutations
Source: Cell Oncol (Dordr). 2022 Aug 5;45(5):913–30. doi: 10.1007/s13402-022-00696-3 (PMC9579113; doi:10.1007/s13402-022-00696-3)
Supplement: Supplementary file 3 — Supplementary file3 (DOCX 26 KB) [file 13402_2022_696_MOESM3_ESM.docx]

**Supplementary Information**

**Materials and Methods**

**MTT analysis**

The 3-(4,5-dimethyl-2-thiazolyl)-2,5-diphenyl-2H-tetrazolium bromide methylthiazolyldiphenyl-tetrazolium bromide (MTT, Sigma-Aldrich, M5655) assay was used as an indirect measure of cell viability. For MTT assays, cells were seeded in 96-well plates overnight and treated with drugs for 24 h at 37 °C with 5% CO_2_. After incubation, cell viability was determined by adding 100 μl MTT solution (5 mg/ml in phosphate-buffered saline (PBS)) to wells followed by incubation for 4 h at 37 °C with 5% CO_2_. MTT mixtures were removed and 100 μl DMSO was added to wells. Samples were shaken for 30 min, and absorbance at 540 nm was recorded for ELISAs. Cell viability was calculated as follows: 1 − average absorbance of treated group/average absorbance of the control group.

**Immunoblotting, immunoprecipitation, immunohistochemistry and immunofluorescence assays**

Immunoblotting and immunoprecipitation were performed as described previously (27, 28).

For immunofluorescence (IF) assays, cells were plated in 8-well chamber slides (SPL, Pocheon-si, Gyeonggi-do, Korea) in advance. When the cells reached 80% confluence, they were washed with PBS and fixed with 4% paraformaldehyde for 20 min at room temperature. Cells were then washed twice with PBS. After blocking with a blocking solution consisting of 5% blocking one-P (Nacalai Tesque, Kyoto, Japan) and 0.1% triton-X in PBS, cells were washed again twice with PBS. The primary antibody in dilution buffer (blocking solution diluted 2 times with PBS) was applied to the cells and incubated overnight at 4°C. After washing the cells with PBS three times, the secondary antibody in dilution buffer was added and incubated for 1 hr at room temperature. A total of 0.1 μg/mL of DAPI was added to the samples and left for 10 min to stain the nuclei. Cells were finally washed three times, treated with Dako mounting solution (Agilent, Santa Clara, CA, USA), and sealed with a cover glass. Images were taken using an apotome laser-scanning microscope and analyzed with Zen Pro software (Carl Zeiss Co. Ltd., Jena, Germany).

For immunohistochemistry (IHC) in tissue sections, slides were placed in citric acid buffer (pH 6.0) and heated at 100 °C for 20 mins for antigen retrieval. Slides were incubated overnight at 4°C with antibodies. Slides were then incubated with an avidin-biotin peroxidase complex (ABC kit, Vector Laboratories, CA, USA) and developed using 3,3′-diaminobenzidine tetrachloride (DAB; Zymed Laboratories, South San Francisco, CA, USA).

For IF in tissue sections, slides were placed in citric acid buffer (pH 6.0) and heated at 100 °C for 20 min for antigen retrieval. Slides were co-immunostained with appropriate antibody overnight at 4 °C. The slides were incubated with Alexa 568-labeled anti-rabbit (1:500) and Alexa 488-labeled anti-mouse (1:500) secondary antibodies. The nucleus was counterstained with DAPI (Sigma-Aldrich) (1:10000), and the stained cells were imaged using a Zeiss Apotome (Carl Zeiss). Quantification of images was measured with an image analyzer (ImageJ, NIH, Bethesda, MD, USA). All statistical analyses of images were performed using GraphPad Prism software 5.0. For immunofluorescence (IF) in human cancer tissue microarray sections, tissue sections stained with appropriate antibody were incubated with appropriate fluorescent secondary antibodies and counterstained with DAPI. Images were viewed under a Zeiss Apotome (Carl Zeiss). Human lung cancer tissue slides were purchased from US Biomax. The antibodies used in these experiments are listed in **Table S1.**

**Subcellular fractionation**

The cytoplasmic and nuclear fractions of all cells were collected using NE-PER Nuclear and Cytoplasmic Extraction reagents (Thermo Scientific, Waltham, MA, USA). Sample preparation was made using the manufacturer’s protocol. The final protein concentration of each sample was measured using a BCA protein assay kit (Thermo Scientific). Equal amounts of protein were applied for western blot analysis to evaluate changes in the subcellular localization of each protein of interest. Lamin A/C was used as the loading control for the nuclear fraction, and beta-actin was used for the cytoplasmic fraction.

**Figure Legends**

**Supplementary Figure S1. HSP27 played a prognostic role in EGFR expressed adenocarcinoma**. (A) Distribution of *HSPB1* gene expression levels in 935 NSCLC patients. (B) Changes in protein expression in NSCLC cell lines by indicated concentration of gefitinib (Gef) treatment (6 hr treatment at indicated concentration). (C) Relative band density of p-STAT3 was expressed as the fold change relative to Gef 0 µM. Protein levels were quantified using ImageJ software.

**Supplementary Figure S2. Phosphorylation and stability of HSP27 were increased in Gef-resistant cell lines.** (A) Comparison of the basal protein expression between Gef-sensitive cells (PC9) and Gef-resistant cells (PC9GR) were detected by western blotting and direct interactions between AKT or p-AKT and HSP27 or p-HSP27 were confirmed using immunoprecipitation. (B) The difference of colocalization between pAKT and HSP27 in PC9 and PC9GR cells using immunofluorescence. The merged area intensity of HSP27 and pAKT were quantified using ImageJ software. Student’s t-test, **p < 0.05*. (C) Independent changes in the cytoplasmic and nuclear expression of Gef-sensitive cells (HCC827) and Gef-resistant cells (H1650) were confirmed using western blotting. (D) Comparison of the basal protein expression between Gef-sensitive cells (HCC827) and Gef-resistant cells (H1650) were detected by western blotting. (E) Changes in protein expression in H1650 cells by low concentration of Gef treatment (6 hr treatment at indicated concentration). (F) Changes in Flag stability according to Flag-tagged HSP27 AAA (HSP27 phospho defective mutant at Ser15, Ser78, and Ser82) or DDD (HSP27 phospho mimicking mutant at Ser15, Ser78, and Ser82) transfection were assessed in HEK293T cells by cycloheximide (CHX) (100 μg/ml) treatment. Band density was expressed as the fold change relative to the control. Results are the means and standard deviations of three independent experiments (**p<0.05*). (G) Changes of direct protein interaction between HSP27 and EGFR or HSP27 and p-AKT or AKT were confirmed using immunoprecipitation after incubation with 100 nM Gef for 6 hr.

**Supplementary Figure S3. HSP27 interacted with EGFR or pAKT according to Gef-sensitive or resistant cells.**

(A) PC9 and NCI-H1650 cells were transfected with 30 nM of siEGFR or siAKT. After transfection, interactions of HSP27 with EGFR or pAKT were analyzed by immunoprecipitation. (B) Proximity ligation assay of HSP27: pAKT and HSP27: EGFR in transfection of siCont and siEGFR or siAKT in HCC827 or H1650 and detect interaction (red dots). Quantification of red dots staining was performed by ImageJ. Graphs are the means and S.D. of three independent experiments. Student’s t-test, **p<0.05*.

**Supplementary Figure S4. Sensitization effect of Gef-resistant cells using HSP27 or AKT inhibitors.**

(A) Structure of J2 (upper). NCI-H1650 and A549 cells were treated with J2 for 24 hr with or without 20 mM N-acetyl cysteine (NAC) and were analyzed by Western blotting (bottom). (B) The effect of co-treatment with AZD5363 (AKT inhibitor) and Gef was assessed in H1650 after 24 hr incubation.

**Supplementary Figure S5. HSP27 inhibition sensitized NSCLC cells regardless of EGFR mutation.** (A) H1650 and H820 cells were treated with Gef at different concentrations (24 hr) and cell lysates were assessed by western blot analysis. (B) NCI-H460 (H460) and A549 cells were treated with Gef at different concentrations (24 hr incubation) and cell lysates were detected by western blot analysis. Fold represents band density relative to control (left). The effect of co-treatment with J2 and Gef was assessed in EGFR wt cell lines after 24 hr incubation (right). (C) Cell survival was determined by the MTT assay after 24 hr of treatment. Results are the means and standard deviations of three independent experiments (**p<0.05*). (D) A549 cell was transfected with siAKT or siHSP27 or J2 and treated with Gef (24 hr incubation) and cell lysates were detected by western blot analysis

**Supplementary Figure S6. HSP27 inhibition overcame gefitinib-mediated resistance in an NSCLC xenograft mouse system.** (A) Immunohistochemistry (IHC) images of CD3 from the tumors just described. IHC scores were calculated using ImageJ software. Student’s t-test, **p<0.05*. (B) Representative immuno-histochemistry images of Ki67, HSP27, and pAKT from the H1650 or A549 xenograft tumors. (C) HSP27 and p-AKT levels in a lung adenocarcinoma tissue. Immunofluorescence of tissue microarrays slides (n=75). In all images colors are as follows: HSP27 (red), p-AKT (green), Merge (HSP27 and p-AKT), and DAPI (blue). All images are 100x magnification (upper). Representative images for comparison of HSP27 (red) and p-AKT (green). Images are 200x magnification (bottom).

**Supplementary Table S1. General information of antibodies utilized in the study.**

| Antibody | Vendor | Catalog number | Application |
| --- | --- | --- | --- |
| HSP27 | Cell signaling | #2402 | WB, IP, IF, IHC |
| p-HSP27 (S15) | Cell signaling | #2404s | WB |
| p-HSP27 (S78) | Cell signaling | #2401s | WB |
| p-HSP27 (S82) | Cell signaling | #2405s | WB |
| AKT | Cell signaling | #4691 | WB |
| p-AKT (S473) | Cell signaling | #4060 | WB, IF, IHC |
| EGFR | Cell signaling | #2232 | WB |
| p-EGFR | Cell signaling | #3777 | WB |
| STAT3 | Cell signaling | #9139 | WB |
| p-STAT3 (Y705) | Cell signaling | #9131 | WB |
| PRAS40 | Cell signaling | #2610 | WB |
| p-PRAS40 (T246) | Cell signaling | sc-7985 | WB |
| MK2 | Cell signaling | #3042 | WB |
| p-MK2 (T334) | Cell signaling | #3041 | WB |
| p38 | Cell signaling | #9212 | WB |
| p-p38 (T180/Y182) | Cell signaling | #9216 | WB |
| β-actin | Santa cruz | sc-47778 | WB |
| c-PARP | Cell signaling | #9541 | WB |
| FLAG | MBL | M185-3L | WB, IP, IF |
| Lamin A/C | Cell signaling | #2032 | WB |
| Ki67 | Dako | M748 | IHC |
| CD3 | Invitrogen | #PA1-29547 | IHC |
